# DUI Detection From Gait Using a Multichannel 1DCNN-Attention-BiLSTM Framework

**DOI:** 10.1109/access.2025.3636473

**Published:** 2025-11-25

**Authors:** SAMUEL CHIBUOYIM UCHE, EMMANUEL AGU, KRISTIN GRIMONE, DEBRA S. HERMAN, ANA M. ABRANTES, MICHAEL D. STEIN

**Affiliations:** 1 Computer Science Department, Worcester Polytechnic Institute, Worcester, MA 01609, USA; 2 Butler Hospital, Providence, RI 02906, USA; 3 Department of Health Law, Policy and Management, Boston University School of Public Health, Boston, MA 02215, USA

**Keywords:** Accelerometer, alcohol intoxication, blood alcohol content, deep learning, gait analysis, smartphone sensors

## Abstract

Alcohol intoxication increases Blood Alcohol Content (BAC) and impairs cognitive, motor, and psychomotor functions, and contributed to over 30% of motor vehicle fatalities in 2017. Traditional methods for detecting Driving Under Influence (DUI), such as breathalyzers and blood tests, are invasive, require external hardware, and are unsuitable for continuous monitoring. Passive approaches such as gait analysis from smartphone sensor data offer a non-invasive solution for detecting impairment and enhancing road safety. Prior work has explored traditional machine learning (ML) and some Deep Learning (DL) approaches but have limitations such as analyzing on handcrafted features and facing challenges such as class imbalance and gait variability. This paper proposes a novel DL framework for passive alcohol intoxication detection from smartphone accelerometer data. A subject-level pre-processing pipeline was employed to address inter- and intra-subject variability, including stratified splitting, low-pass filtering, sliding window segmentation, and random oversampling to mitigate class imbalance. We propose the Multichannel Hybrid 1D-CNN-Attention-BiLSTM (MC-Hybrid) model, which extracts short-term features via parallel 1D-CNNs, uses a self-attention mechanism to increase weights on predictive patterns, and utilizes a bidirectional LSTM to model temporal dependencies. Rigorous evaluation includes comparison to a comprehensive set of ML and DL baselines, investigation of multiple sensor data window sizes, attention types, and ablation studies. MC-Hybrid achieved 93% accuracy and an F1-score of 0.8653, outperforming the state-of-the-art by 9.5% and all baselines by 9.0%. Self-attention resulted in a 2% performance gain over other attention mechanisms, demonstrating its effectiveness in DUI detection. The proposed method could be a practical, non-invasive approach to detect alcohol impairment.

## I. INTRODUCTION

### A. MOTIVATION

Alcohol crosses the blood-brain barrier within minutes of consumption, raising Blood Alcohol Content (BAC) [1] and impairing neuromotor, cognitive, and psychomotor functions [2], [3], [4], causing longer reaction times [5]. Furthermore, Alcohol intoxication affects consciousness, perception, judgment, behavior, and balance [3], with severe cases (BAC ≥ 0. 45%) being life-threatening. The Center for Disease Control (CDC) reports that annually, 178,000 deaths in the US are due to excessive alcohol use [6]. Driving under the influence (DUI) of alcohol contributes significantly to traffic accidents, with intoxicated drivers fifteen times more likely to collide with other drivers [7], [8]. In fact, in 2022, 32% of all traffic deaths were alcohol-related, with 13,524 fatalities recorded [9], [10]. On average, 34 alcohol-related deaths occur daily, or one every 42 minutes [11], with the estimated economic cost, including medical bills, legal fees, and lost productivity totaling up to $123.3 billion annually [12]. These statistics highlight the urgent need for novel methods to detect alcohol intoxication to enhance road safety.

Traditional BAC detection relies on breathalyzers, transdermal alcohol monitors (TAMs), and blood tests. Although accurate, these methods which require purchasing and carrying additional hardware, are costly, intrusive, and impractical for widespread or DUI enforcement at roadside. This underscores the need for passive, non-invasive solutions that unobtrusively detect alcohol-induced impairments to reduce DUI incidents and improve road safety.

### B. BACKGROUND

Gait— a semi-automatic motor function that requires attention [13], [14]— is affected by alcohol intoxication, making it a viable non-invasive detection modality. Intoxicated gait exhibits increased trunk sway, altered kinetics, kinematics, and changes in velocity, balance, cadence, and stride attributes [2]. Severe intoxication can cause gait ataxia, cerebellar damage, white matter degradation, and reduced striatal volumes [15], [16]. Law enforcement currently relies on roadside Field Sobriety Tests (e.g., walk-and-turn, one-leg stand) to assess DUI based on gait sway, inability to walk straight, or difficulty walking heel-to-heel [17]. Research has shown that mild (BAC 0.01%–0.1%) and moderate (BAC 0.15%–0.3%) intoxication affect postural stability, coordination, and psychomotor skills, increasing body sway on YZ (anterior-posterior) and XY (medio-lateral) planes [3], [18], [19].

### C. PRIOR WORK

Machine learning (ML)-based gait analysis has successfully detected conditions such as Parkinson’s disease [20], Alzheimer’s disease [21], and stroke rehabilitation [22]. Specific to smartphone-based alcohol intoxication detection, Arnold et al. classified manually engineered features from smartphone accelerometer data, achieving 70% accuracy [23]. Their work extracted and classified features such as Signal-to-Noise-Ratio (SNR) [24], cadence, skewness, and kurtosis [25]. Aeillo and Agu improved accuracy to 89.45% by incorporating postural sway features [26], but analyzed data that was collected from subjects wearing drunk buster goggles rather than actual alcohol consumption. ML methods based on such data may fail to generalize to real-world scenarios involving real alcohol consumption [27], [28]. Both studies used traditional machine learning algorithms (Random Forest and J48) with manual feature extraction, which is tedious, error prone, and requires domain expertise.

Li et al. [29] proposed a deep learning (DL) approach, encoding time series data into Gramian Angular Field (GAF) images that were classified into BAC levels using a bilinear convolutional neural network(CNN). Their study achieved 83.5% accuracy. Table 1 summarizes these approaches. For regression-based alcohol intoxication estimation, Gharani et al. [30] achieved 0.0226 Root Mean Square Error (RMSE) using a MultiLayer Perceptron (MLP), while Li et al. [31] used Bidirectional Long Short Term Memory (Bi-LSTM) and CNN, reaching RMSEs of 0.0167 and 0.0168 respectively. Table 2 outlines these methods. More broadly, other intoxication detection approaches include camera-based [32], pressure sensor-based [33], [34], and Inertial Measurement Unit (IMU)-based gait analysis, which all have limitations. Camera-based methods suffer from illumination and angle dependencies, while pressure-based systems lack portability, and IMU-based approaches require body-worn sensors. This study proposes an IMU-based, passive, unobtrusive DUI asssessment from gait but utilizes data gathered from the subject’s smartphone sensors.

### D. SPECIFIC PROBLEM

Our objective is to propose a novel deep neural networks framework for detecting alcohol-induced gait impairment from smartphone inertial sensor data. We formulated our study as a binary classification problem.

### E. CHALLENGES

Challenges that make achieving this objective difficult include: (1) inter-subject variability in alcohol effects caused by differences in body weight, stride length, alcohol tolerance, and metabolism complicates ML model generalization [35]. (2) Sensor variability across smartphone models, device orientation, and placements–handheld, pocketed, or bagged– affects data quality. (3) Dataset imbalance – our NIH-funded dataset had 18 million samples from 121 subjects, but had 14 million sober samples vs. 4 million impaired samples, creating class imbalance. (4) Fine-grained classification– gait signals belonging to the sober and intoxicated classes may appear similar as shown in Fig. 2, making classification challenging. (5) Confounding factors such as stress, injuries, and fatigue can impact gait, complicating attribution of impairment to intoxication. Addressing these challenges require advanced signal processing and innovative machine learning techniques to achieve clinically usable accuracy on smartphone-based alcohol intoxication detection.

### F. OUR APPROACH

We propose a DL framework that incorporates a multichannel (parallel) 1D-CNN to extract short-term fine-grained spatial-temporal features from raw smartphone accelerometer gait data. Parallel BiLSTM layers are utilized to model long-term gait dependencies in this architecture, and parallel self-attention layers focus on the most predictive temporal features. Furthermore, we design a subject-level stratified data pre-processing pipeline that included noise filtering, windowing, class upsampling, five-fold cross-validation and ablation studies to enhance generalization and robustness. To the best of our knowledge, this is the first study to apply this integrated pipeline and architecture on a large-scale, real-world, NIH-funded dataset of alcohol-impaired gait using actual consumption, not simulated effects.

**FIGURE 1.**
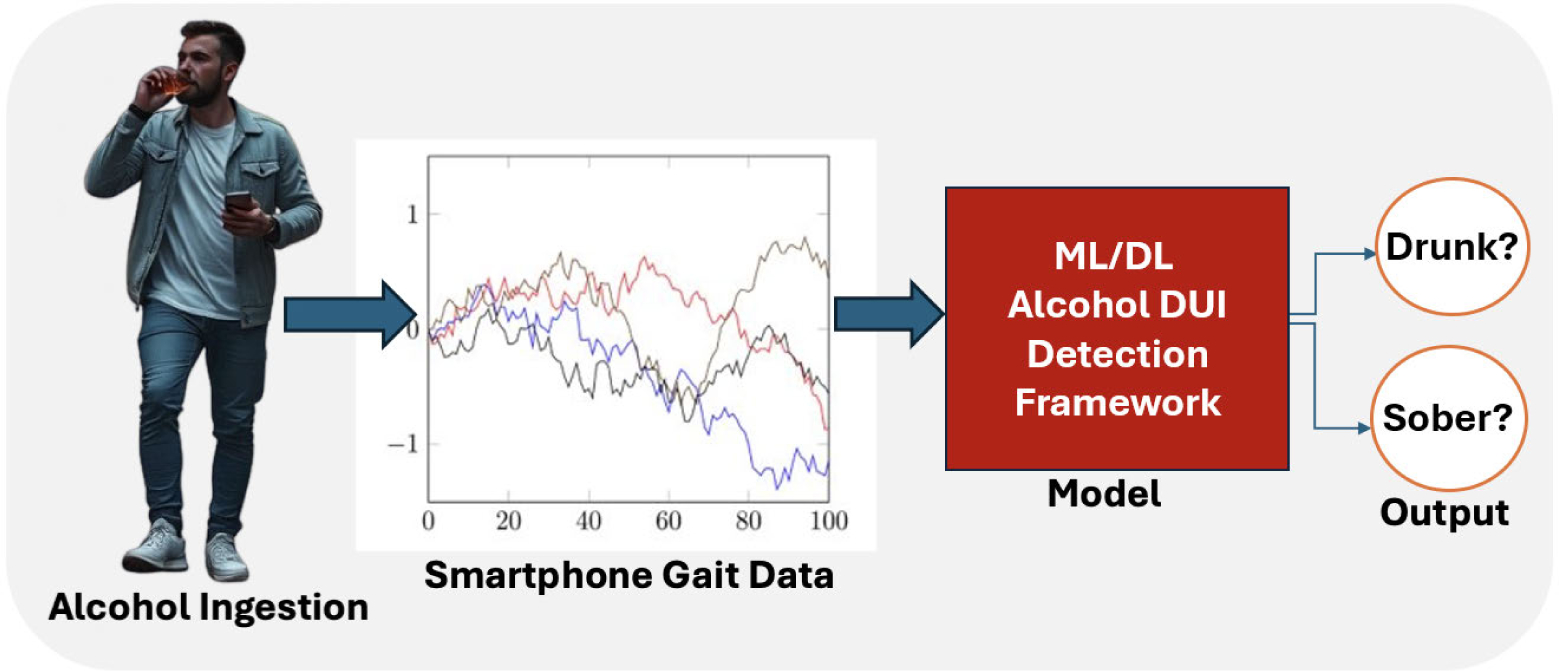
High level Scenario of alcohol detection from gait.

**FIGURE 2.**
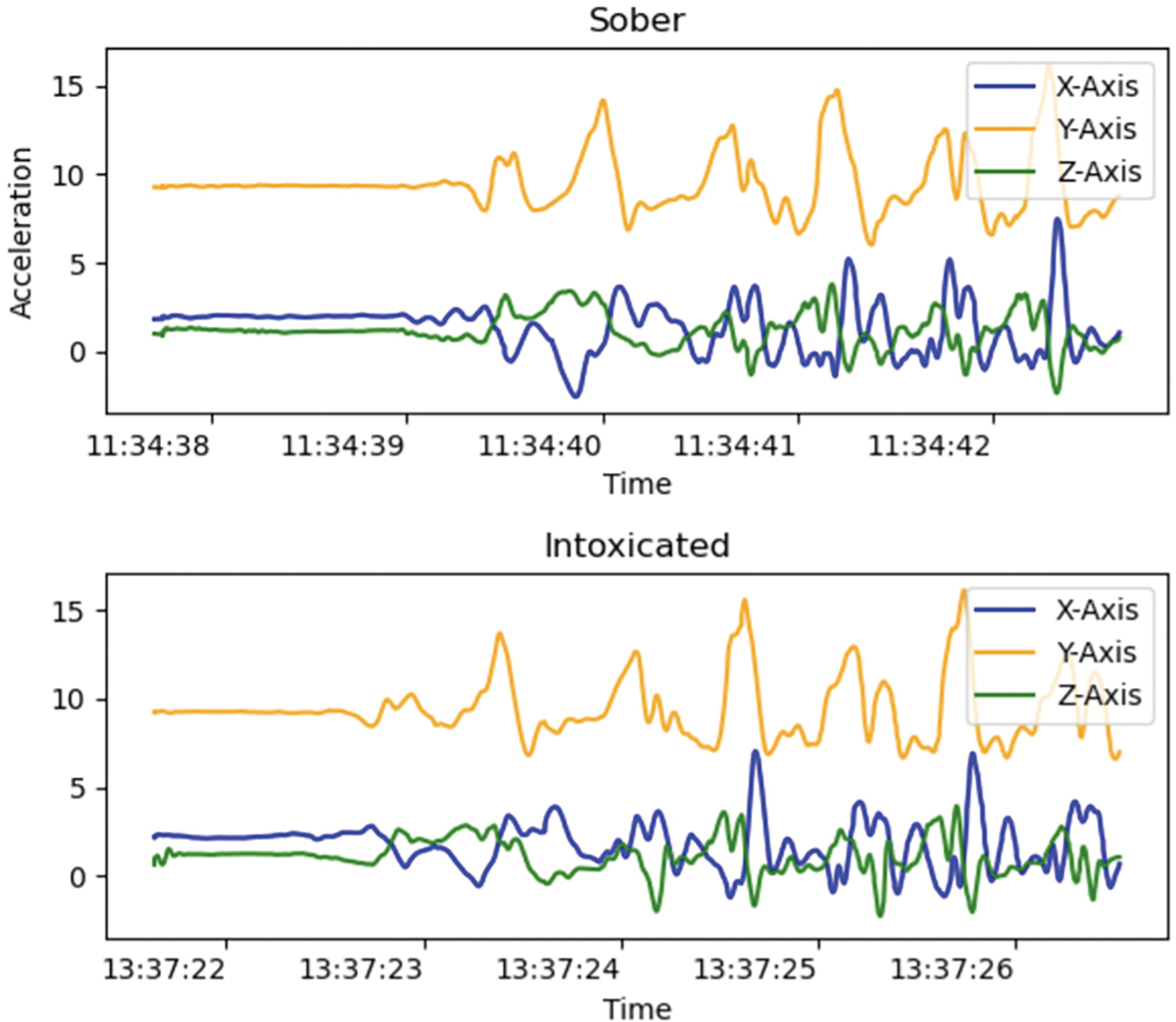
Fine-grained sensor signal problem.

Fig. 1 illustrates our machine learning framework for analyzing gait patterns from smartphone sensor data which are indicative of alcohol intoxication We propose this approach leveraging the smartphone’s ubiquity (owned by 98% of Americans [36]) and its ability to passively collect movement data from its built-in sensors. In the real world, this system passively monitors users, detects intoxication, and could be used to trigger innovative safety interventions such as notifying the smartphone owner once they are over the legal limit, immobilizing their vehicle or hailing a taxi to transport them safely. This non-intrusive approach detects alcohol-induced psychomotor impairments in real time, potentially preventing DUI incidents and injuries [37]. However, it is instructive to note that our proposed system has not been evaluated yet on individuals with pre-existing gait abnormalities resembling intoxicated gait, a direction planned for future work.

### G. CONTRIBUTIONS

#### Contributions of this paper include:

1. *Custom, subject-level sensor data pre-processing pipeline:* to handle the unique challenges of smartphone gait data in DUI detection. This includes subject-level normalization to mitigate inter- and intra-subject variability, outlier removal, denoising with low-pass filtering, and a 50% overlapping sliding window segmentation to ensure temporal continuity. To address the highly imbalanced nature of intoxicated samples, we applied subject-level random oversampling–a first in this context– which significantly enhanced generalization while preserving class balance across stratified train, validation, and test splits.
2. *Novel Multichannel 1D-CNN-Attention-BiLSTM (MC-Hybrid) architecture for DUI detection:* This hybrid deep learning architecture integrates multichannel temporal feature extraction via parallel 1D-CNN layers, critical pattern localization through parallel self-attention layers, and sequential modeling using parallel Bi-LSTM layers. Unlike existing methods, our framework simultaneously processes X, Y, Z gait channels in parallel, allowing it to learn spatial-temporal dependencies specific to intoxicated movement patterns. This architecture and the components it integrates have not previously been applied to alcohol DUI gait classification.
3. *Comprehensive systematic benchmarking against deep learning and traditional baseline models:* (Random Forest, XGBoost, Naive Bayes, Decision Tree, Logistic Regression) that analyzed handcrafted features. Baseline deep learning architectures included LSTM, 1D-CNN, BiLSTM and LSTM-GRU. To the best of our knowledge, this is the first study to benchmark such a wide range of models specifically for alcohol intoxication detection from smartphone-based gait data.
4. *Systematic investigation of multiple attention mechanisms and window sizes*
5. *Systematic interpretability analyses* was conducted via attention weight visualization of MC-Hybrid’s multi-channel architecture, revealing temporal discrimination patterns. Channel ablation was utilized to quantify per-channel contributions and phase-wise analysis identified critical time segments.

### H. SIGNIFICANCE

The proposed MC-Hybrid DL framework for smartphone-based DUI assessment from gait data could improve real-time, passive alcohol intoxication detection, reduce DUI incidents, and improve public safety.

The rest of this paper is as follows. Our alcohol gait intoxication dataset, preprocessing pipeline, and deep learning architecture are introduced in section II. Our evaluation and experimental results are presented in sections III and IV respectively. Our results are discussed in section V, and limitations and areas of improvement are presented in section VI. Finally, sections VII and VII present our conclusions and future work, respectively.

## II. METHODOLOGY

### A. DATASET

**FIGURE 3.**
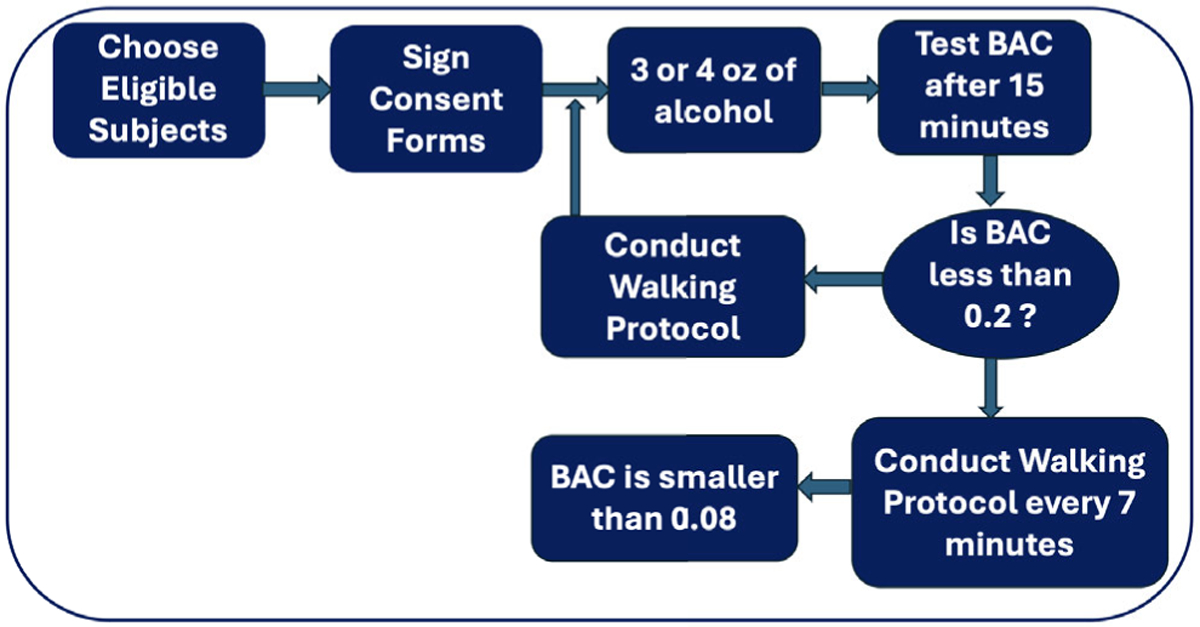
Alcohol administration protocol.

This study analyzed gait data collected from 121 participants in an Institutional Review Board (IRB) approved study (reference number 1612–001) conducted at Butler Hospital. The study was designed by experts in alcohol experimentation. As shown in Fig. 3, during data collection, participants underwent structured alcohol administration protocols, repeated walking tasks with smartphone strapped to their waist, and provided ground truth BAC measurements using breath alcohol analyzers. During the walk tasks along a 75-foot hospital corridor, gait data from the accelerometer and gyroscope sensors were passively captured via a smartphone app. It is instructive to note that in this study, we focused on the three-axis accelerometer signals collected from waist-mounted smartphones, as they capture the periodic acceleration patterns of gait in the vertical, mediolateral, and anteroposterior directions reliably. While gyroscope signals provide angular acceleration and orientation information, prior work has demonstrated that accelerometer-derived features alone are sufficient to detect alcohol-related gait impairments [38], [39], [40]. Utilizing only accelerometer data also reduces computational complexity and energy consumption, making our approach more feasible for continuous mobile usage. Comprehensive details on participant recruitment, study procedures, and data collection are provided in the Appendixes VIII section.

### B. DATA PREPROCESSING

**FIGURE 4.**
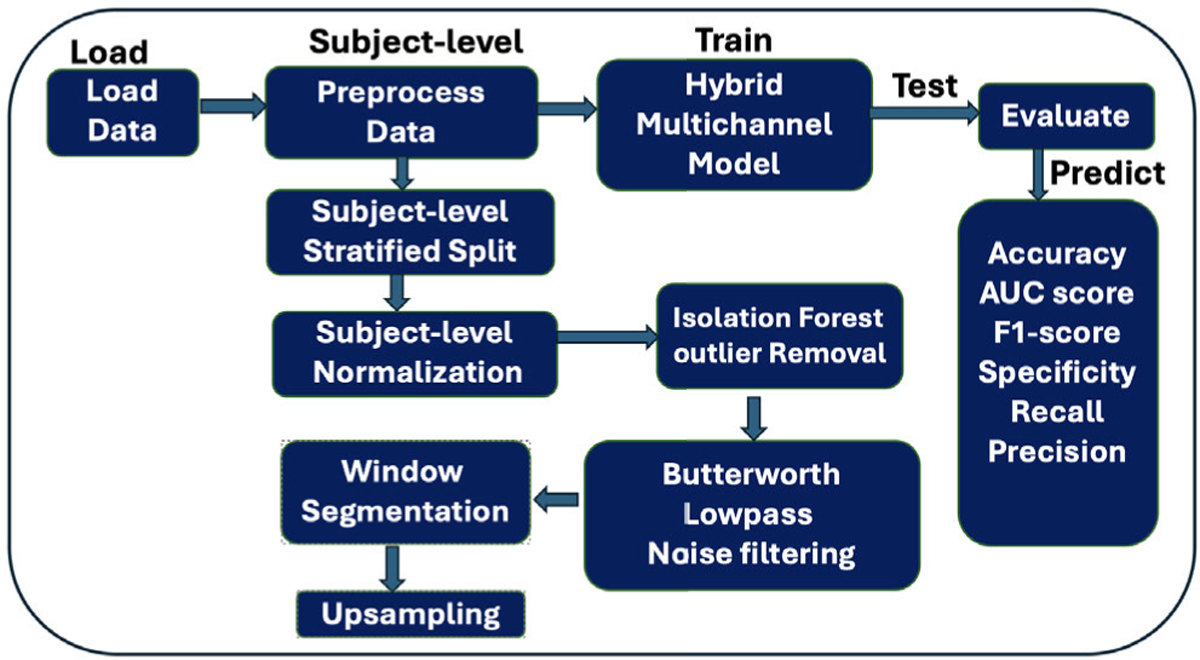
Alcohol intoxication detection pipeline.

As shown in Fig. 4, our analysis involved an innovative set of subject-wise, pre-processing steps that generated high quality data suitable for supervised machine learning. Pre-processing steps included stratified split, normalization, outlier removal, noise filtering, sliding window segmentation, and oversampling of the train set.

We cleaned our data to handle missing values, and dropped duplicate data samples. We split our data into 70–20-10 train-test-validation sets using a stratified subject-wise approach while ensuring equal distribution of data in classes across splits. With an already high class imbalance given by fewer intoxicated samples as shown in Table 3, this approach mitigates potential data leakage.

To account for inter- and intra-subject variability in alcohol intoxication, the remaining pre-processing steps were performed at the subject level. Within each data split, each subject’s data was Z-score normalized independently, ensuring that each data channel (X, Y, Z) had a mean of 0 and a standard deviation of 1. This standardization helps normalize feature scales across subjects. Equation (1) expresses z-score normalization.

(1) $$z=\frac{x\;-\;\mu }{\sigma }$$

To remove outliers (falls or 180-degree turns) that may occur during normal walk, we utilized the Isolation Forest outlier removal algorithm. Isolation Forest is an unsupervised anomaly detection algorithm that identifies anomalies by isolating data points using randomly constructed decision trees. The anomaly scores are computed using (2),

(2) $$s(x,n)=2^{-\frac{E(h(x))}{c(n)}}$$

where average path length (c(n)) or normalization factor for the dataset of size n is given by (3)

(3) $$c(n)=2\cdot H(n-1)-\frac{2(n\;-\;1)}{n},\;H(i)=\sum_{k=1}^{i}\frac{1}{k}$$

Through rigorous experimentation, we determined that a contamination ratio (proportion of the dataset that are considered outliers) of 0.05 yielded the best outlier removal results. A third-order Butterworth low-pass filter with a cutoff frequency of 20 Hz and a sampling rate of 210 Hz was designed to remove high-frequency noise while preserving lower-frequency signal components for each subject; the filter operates by applying forward and backward filtering to the X, Y, and Z axes individually, ensuring phase distortion is minimized. Equation (4) is the expression for low-pass filtering.

(4) $$H(s)=\frac{1}{\sqrt{1+{\left(\frac{s}{\omega_{c}}\right)}^{2n}}},\;\omega_{c}=2\pi \;\times \;\text{cutoff}\;\text{frequency}$$

For each subject, the cleaned data was then segmented into fixed-sized windows using a sliding window of size 1024 ( 4.88 seconds) with a 50% overlap. After evaluating window sizes of 256, 512 and 1024, 1024 was discovered experimentally to be optimal given our sampling rate. Our experimental findings were supported by those in prior work where slightly longer segments were used, as intoxicated subjects tended to walk carefully and take slower steps as their BAC increases [31]. To ensure that our methodology was not biased towards the majority sober class, an oversampling algorithm was used to upsample the number of intoxicated samples in the training set to match the number of sober samples.

### C. MULTICHANNEL 1D-CNN ATTENTION BI-LSTM FRAMEWORK

**FIGURE 5.**
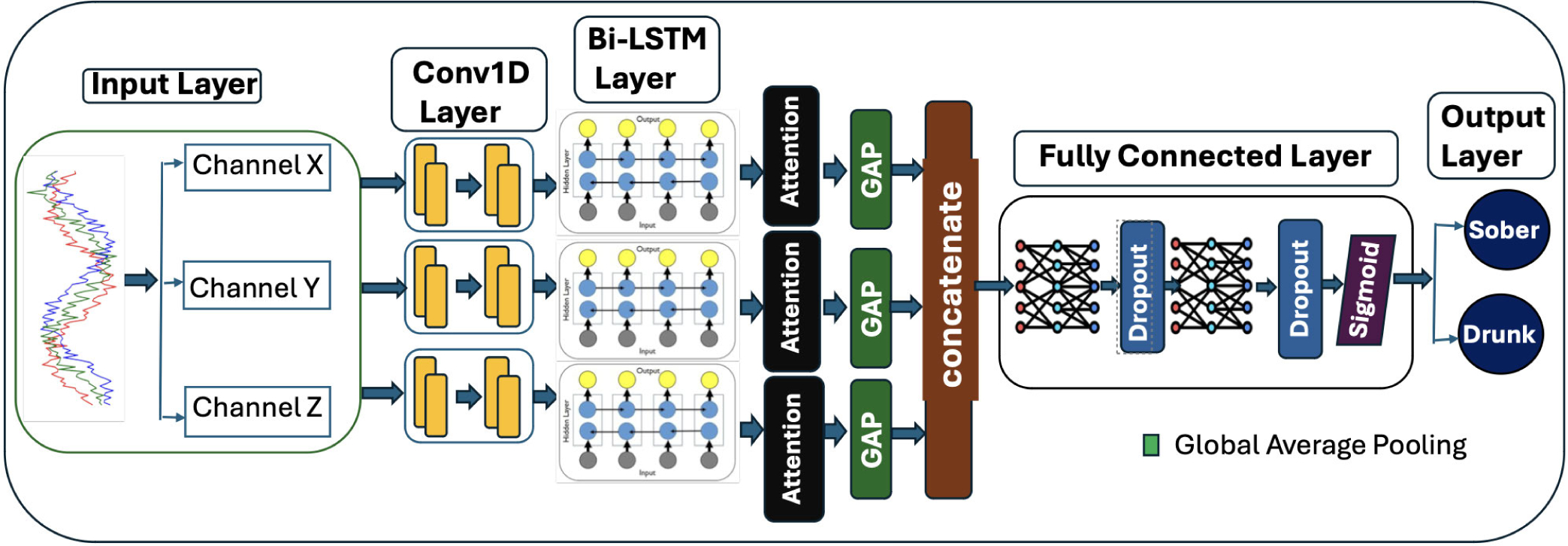
Proposed multichannel 1DCNN-attention-biLSTM (MC-Hybrid) architecture for alcohol intoxication detection.

This paper proposes a novel multichannel hybrid framework shown in Fig. 5 that comprises of several parallel layers where each accelerometer axis (X, Y, Z) is processed independently using Conv1D-BiLSTM-Attention layers. The framework has a feature extraction layer, a fully connected layer, and an output layer. Parallel input, conv 1D, BiLSTM, attention and global average pooling layers make up the feature extraction layer. This design allows axis-specific temporal patterns to be learned before integration, improving feature extraction from multi-dimensional gait signals.

#### 1) INPUT LAYER

At this layer, the segmented data from the X,Y,Z axes are split into three channels to be fed in parallel into the feature extraction layers.

Mathematically, the input sequence is given as

(5) $$X_{\text{channel}\;}=x_{1},x_{2},\ldots ,x_{T},\;X_{\text{channel}\;}\in R^{T}.$$

#### 2) conv 1D LAYER

By applying a sliding filter ω over the input, the Conv 1D layer extracts local temporal gait features, such as variations in step length and cadence due to intoxication. For each input channel, two Conv 1D layers were utilized; the first captures low-level patterns, while the second refines them. The convolution operation, followed by a ReLU activation function, is expressed as:

(6) $$\begin{array}{l} y_{k}^{\left(c\right)}=\sigma \left(\sum_{i=1}^{K}\;w_{i}^{\left(c\right)}\cdot x_{t+k-i}^{\left(c\right)}+b^{\left(c\right)}\right),k\in [1,T],\\ c\in \{X,Y,Z\} \end{array}$$

The ReLU function is defined as:

(7) σ(z)=max(0,z)

#### 3) BIDIRECTIONAL LSTM

A parallel Bi-directional Long Short Term Memory (Bi-LSTM) was used to capture long-term temporal relationships in our input sequence. This layer enabled our framework to recognize gait changes, irregular steps, and balance problems. Bi-LSTMs process sequential data in both forward and backward directions to capture past and future temporal dependencies. To accomplish this, Bi-LSTMs utilize gates and special memory cells. These memory cells help the model retain predictive patterns in gait data, while ignoring noise. We summarize the process with Equation (8):

(8) $$h_{t}^{\left(c\right)}=\left[h_{t}^{\text{forward},(c)},h_{t}^{\text{backward},(c)}\right]\;\;\text{where}\;c\in \{X,Y,Z\}$$

where h_t represents the hidden state combining past and future temporal dependencies.

#### 4) ATTENTION LAYER

Introduced by Vaswani et al. [41], self-attention is crucial to modeling long-range dependencies. In our framework, it emphasizes predictive samples in the gait sequence, which indicate intoxication. Equation (9) summarizes the self-attention mechanism for each channel.

(9) $${\;\text{Output}}_{c}=softmax\left(\frac{X_{c}W_{Q}^{\left(c\right)}{\left(X_{c}W_{K}^{\left(c\right)}\right)}^{⊤}}{\sqrt{d_{k}}}\right)X_{c}W_{V}^{\left(c\right)}\;\text{where}\;c\in \{X,Y,Z\}$$

#### 5) GLOBAL AVERAGE POOLING

The global average pooling layer serves as a dimensionality reduction layer for the gait features extracted from the previous layers. It reduces the sequence to a fixed-length feature vector for each channel, which helped our framework to focus on predictive gait movement information, while filtering out noise. Equation (10) shows average pooling.

(10) $$z_{k}^{\left(c\right)}=\frac{1}{T}\sum_{t=1}^{T}h_{t}^{k,(c)},\;c\in \{X,Y,Z\}$$

- $z_{k}$: Pooled feature for the k-th dimension.
- T: Number of time steps.

After the Global Average Pooling layer, the features extracted from the three channels are concatenated to form a full gait profile as shown in Equation (11).

(11) $$z=\left[z_{\text{channel1}\;},z_{\text{channel}2},z_{\text{channel}3}\right]$$

#### 6) DENSE (FULLY CONNECTED) LAYER

At this layer, our framework learns non-linear combinations of gait features by computing weighted sums of the input features followed by a ReLU activation function. To prevent overfitting and improve generalization, dropout layers are applied after each fully connected layer to permutate the fractions p of neurons active during training. After tuning the dropout rate using Bayesian Optimization, the dropout rate=0.696581562334145 was selected as it achieved the optimal AUC score on the validation set. Equations (12) and (13) express the dense function and dropout layer.

(12) y=σ(W·z+b)

(13) $$h_{i}^{\prime }=\left\{\begin{array}{ll} h_{i} & \;\text{with}\;\text{probability}\;1-p,\\ 0 & \;\text{with}\;\text{probability}\;p. \end{array}\right.$$

The final dense layer produces the final probability of intoxication using a sigmoid activation function shown in (14).

(14) $$\overset{ˆ}{y}=\frac{1}{1\;+\;exp(-z)}$$

Table 4 shows optimal hyperparameter values utilized in our final MC-Hybrid model as determined by Bayesian Optimization. The Adam optimizer and a low learning rate (0.000242) were employed to ensure stable convergence on temporal data. The convolutional filters (117 and 175) and kernel size (6) were selected to balance gait feature extraction from the multichannel input and computational efficiency while capturing local temporal dependencies. The Bi-LSTM layer units (85) modeled long-term sequential dependencies, while the high dropout rate (0.6966) mitigates overfitting, given the model’s complexity. Dense layers with 129 and 188 units enabled higher-level feature abstraction prior to the sigmoid output. These hyperparameter values optimized MC Hybrid model’s performance for DUI detection, ensuring model generalizability.

## III. EVALUATION

### A. EVALUATION METRICS

Our classification metrics include accuracy, which measures the proportion of correctly identified intoxicated and sober cases to the total number of cases evaluated, expressed in 22, F1 score as expressed by 23, and Area under the ROC Curve (AUC-ROC) expressed in 24 that is calculated by summing up the trapezoid area under true positive rate and false positive rate curve. Recall, Specificity and Precision are expressed by Equations (26), (25), and (27) respectively in the Appendixes section.

### B. BASELINE MODELS

The performance of our proposed MC-Hybrid framework was compared with that of the following comprehensive set of baseline ML methods: 1) Support Vector Machine (SVM) identifies optimal decision boundaries for class separation, making it effective for distinguishing sober and intoxicated states. 2) Naïve Bayes uses a probabilistic approach based on Bayes’ theorem, a simple baseline for classification. 3) Random Forest (RF) combines multiple decision trees, handling complex feature interactions well. 4) Decision Trees offer interpretability and handle non-linear relationships algorithm for classification of handcrafted features. 5) The Single-Layer Perceptron (SLP) is a basic neural network for binary tasks, useful for baseline comparisons. 6) K-Nearest Neighbors (KNN) uses instance-based learning to evaluate class separability. 7) Logistic Regression (LOG-REG) is a simple, effective linear model for binary classification. Deep learning baselines include: 1) 1D-CNN: Extracts spatial features from time series data for classification. 2) Bi-LSTM: Captures sequential dependencies in both directions for enhanced pattern recognition. It was included because it achieved good results in similar prior work [31]. 3) LSTM-GRU: Combines Long Short-Term Memory (LSTM) and Gated Recurrent Unit (GRU) for efficient long-term dependency capture. 4) LSTM: Handles sequential data effectively, overcoming vanishing gradient problems in Recurrent Neural Networks (RNNs). It was included because it achieved good results in similar prior work [31].

#### Implementation details:

Our ML models were implemented using the Tensorflow library and trained on NVIDIA Tesla K80, P100, and V100 GPUs. Due to its higher parameter count compared to both traditional ML and DL baselines, the MC-Hybrid model required a longer computation time. For the train-test-val split in our study, training the MC-Hybrid model required approximately one hour, with an average inference time of 150 milliseconds per sample. We trained for 50 epochs while implementing early stopping and learning rate scheduling to avoid overfitting to the training set and to maintain good performance on unseen data. Early stopping is a regularization technique that halts training when the model’s performance on a validation dataset stops improving [42]. During five-fold cross-validation, each fold was trained for 30 epochs with early stopping, yielding an average inference time of 250 milliseconds per sample. In contrast, traditional ML baselines (SVM, Logistic Regression, Naïve Bayes, Decision Tree) trained within seconds with negligible inference times. Despite the additional training overhead, the significant performance improvements of MC-Hybrid in accuracy (93% vs. 84%) and AUC (0.98 vs. 0.88) and its reasonable inference time, justify the trade-off and demonstrate that MC-Hybrid remains computationally feasible for practical binary classification tasks. Overall, based on the inference times for MC-Hybrid, both the traditional split and five-fold cross-validation approaches are suitable for mobile user scenarios. A Binary Cross Entropy (BCE) loss function, expressed in (15), was utilized for both the traditional split and five-fold cross-validation.

(15) Loss=-(ylog(p)+(1-y)log(1-p))

## IV. RESULTS

### A. MC-HYBRID MODEL TRAINING PERFORMANCE

#### 1) DEMONSTRATING NO OVERFITTING BY EXAMINING TRAIN-VALIDATION LOSS CURVES

**FIGURE 7.**
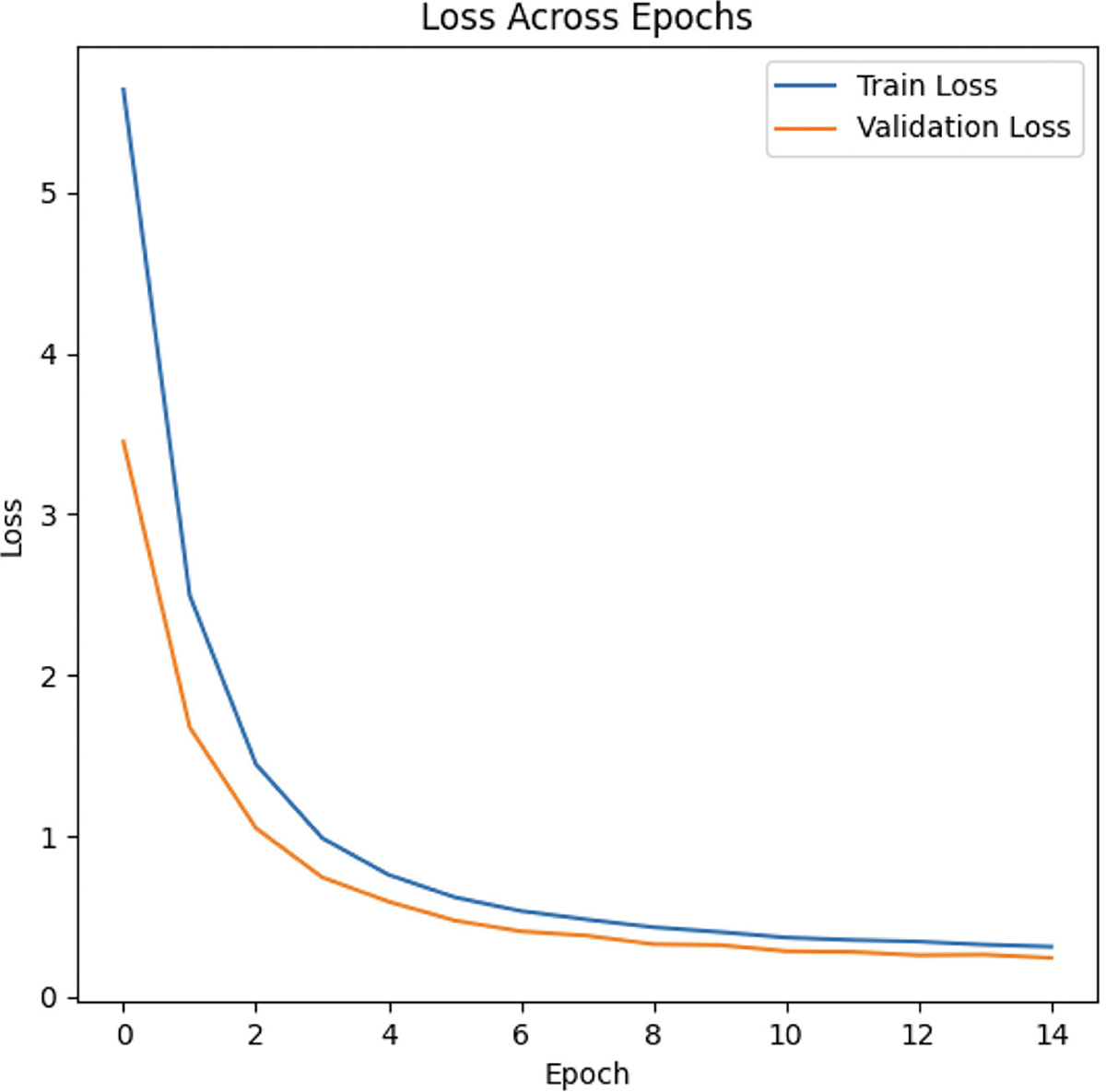
MC Hybrid train-validation loss curve.

Fig. 7 shows that MC-Hybrid’s prediction performance improves over time. A decreasing training loss implies the model is learning patterns in the training data and a decreasing validation loss indicates the model generalizes well to unseen data. The model converges, showing effective learning. After convergence, the difference between our model’s train-validation loss curves is less than 10%, demonstrating that there was minimal overfitting.

#### 2) AUC SCORE PLOT

**FIGURE 8.**
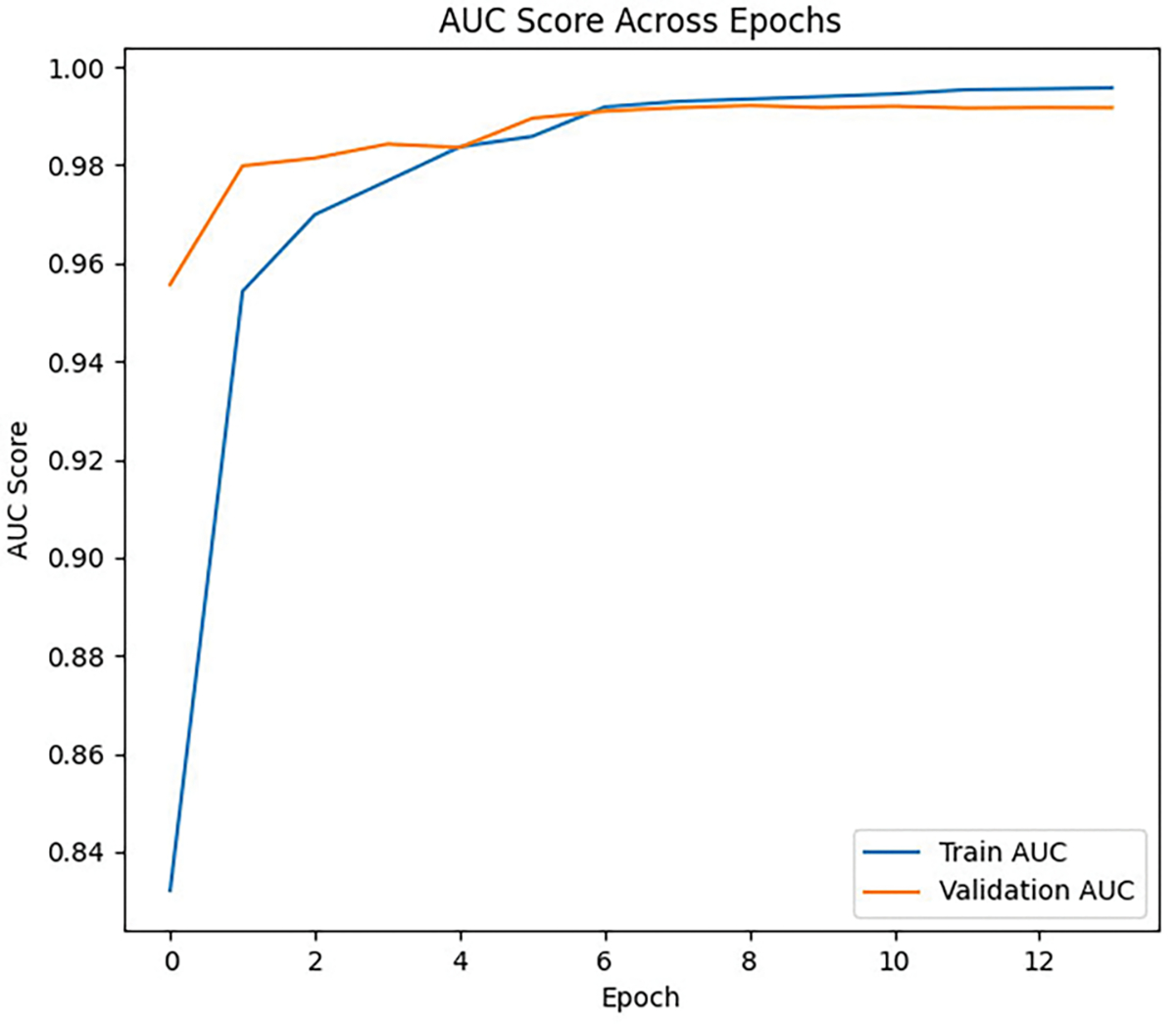
MC-Hybrid AUC train-validation plots.

during training is shown in Fig. 8. AUC reflects the model’s ability to rank positive (intoxicated) examples higher than negative (sober) ones. In our imbalanced dataset, AUC was an effective metric for evaluating model performance, showing how well it differentiates between classes and generalizes to unseen data [43], [44]. The increasing AUC scores for both the training and validation sets indicate improved class separation over epochs.

#### 3) AUC-ROC CURVE

**FIGURE 9.**
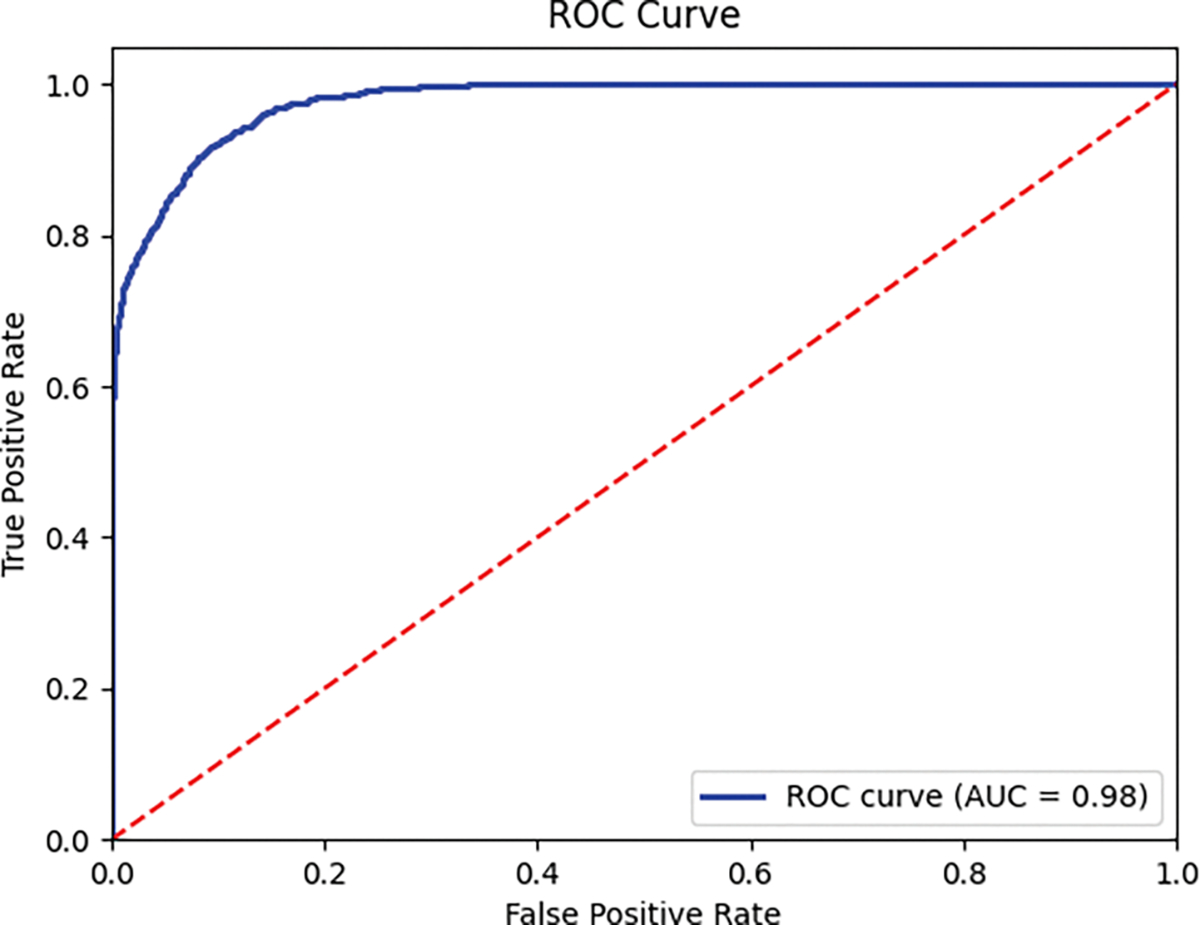
MC-Hybrid AUC-ROC curve.

Fig. 9 presents the ROC curve, a key performance metric for classification. Despite the challenge of distinguishing sober and intoxicated classes, our MC-Hybrid model demonstrated exceptional performance with an AUC score of 0.98, effectively minimizing false positives while accurately detecting true positives.

#### 4) PRECISION-RECALL CURVE

**FIGURE 10.**
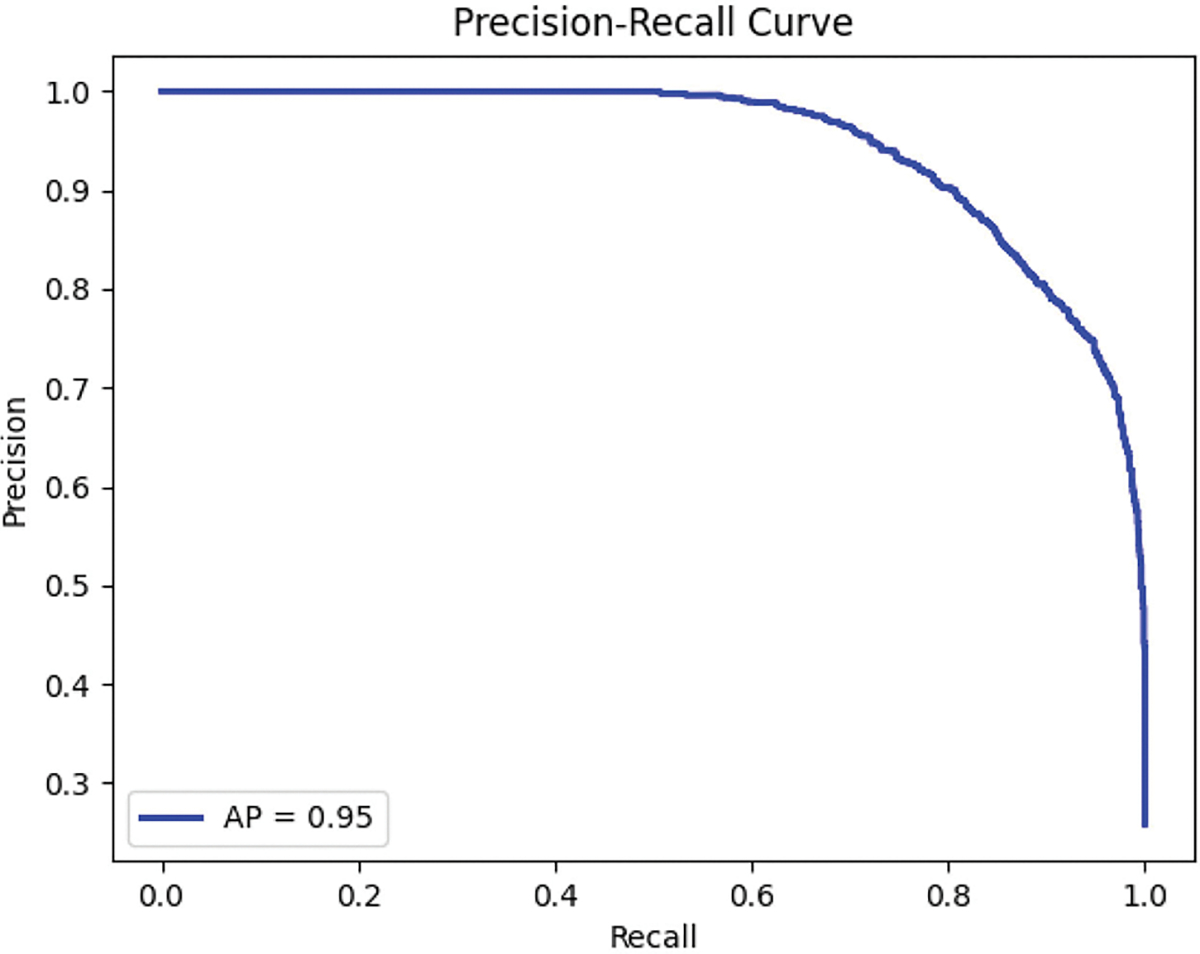
MC-Hybrid Precision-Recall curve.

Fig. 10 shows the precision-recall curve, highlighting the trade-off between precision and recall, crucial for evaluating the imbalanced intoxicated class. The model achieved an average precision of 0.95, indicating strong reliability in positive predictions and robust performance in the intoxicated class.

#### 5) CONFUSION MATRIX

**FIGURE 11.**
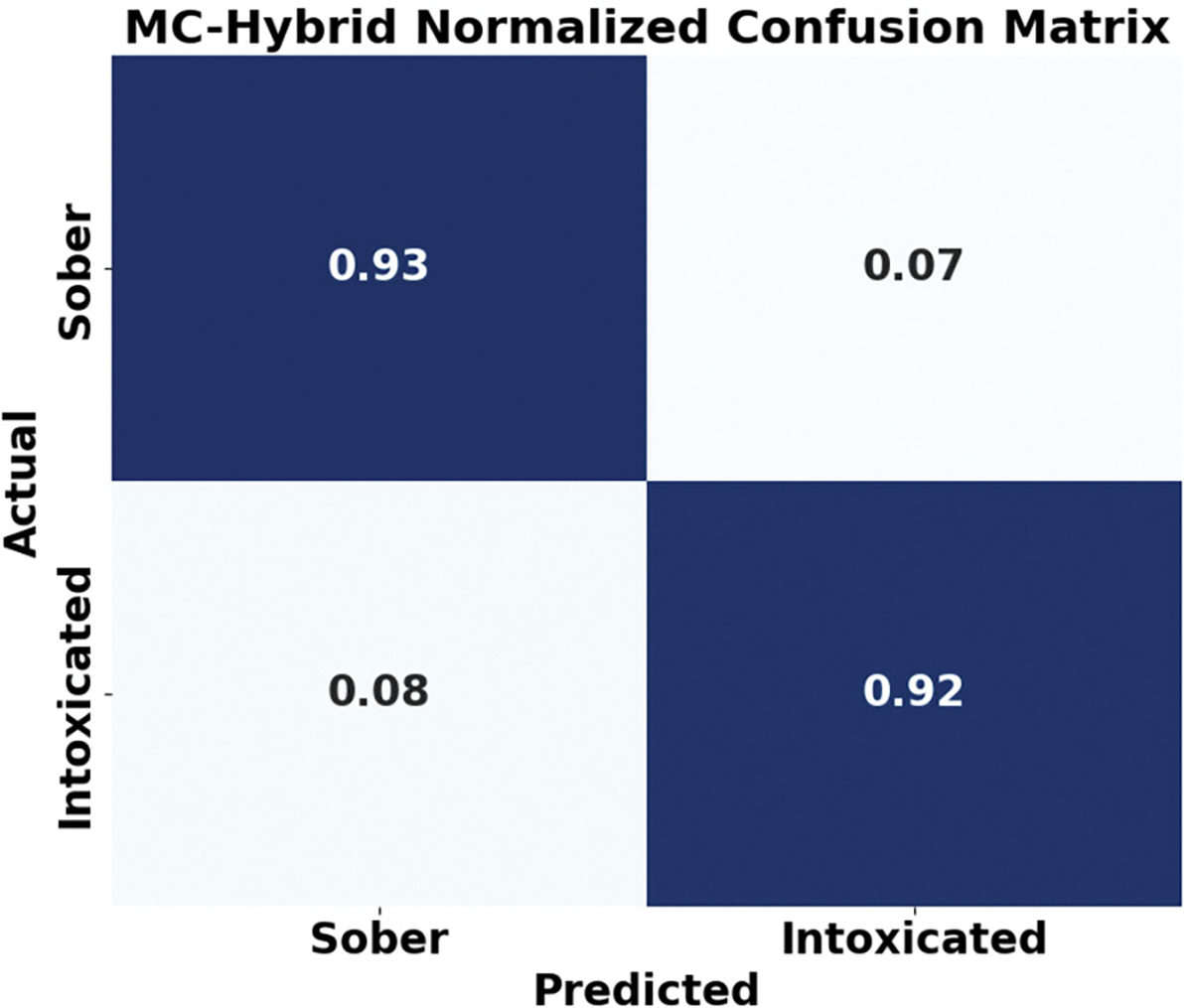
MC-Hybrid confusion matrix.

**FIGURE 12.**
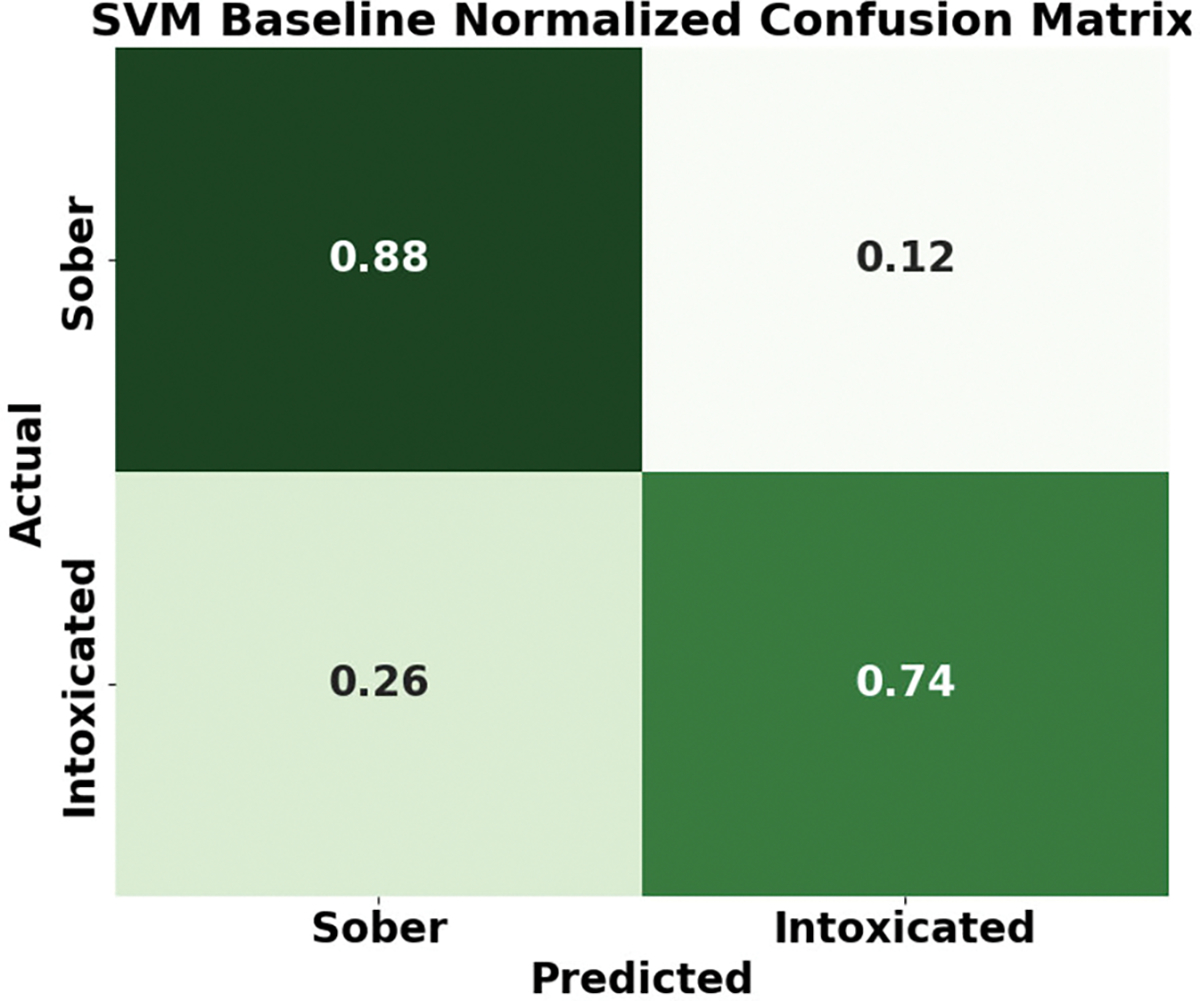
SVM Baseline confusion matrix.

Fig. 11 and Fig. 12 compare the confusion matrices of MC-Hybrid and an SVM baseline. The MC-Hybrid model achieved high accuracy (0.93 and 0.92) with low mis-classification (false negatives: 0.08, false positives: 0.07). In contrast, the SVM model showed a higher false negative rate (26%). These results demonstrate MC-Hybrid’s superior classification capability.

### B. MC-HYBRID VS PRIOR APPROACHES

We compared the performance of MC-Hybrid with previous classification methods of alcohol intoxication. With a 93% accuracy and F1 score of 0.8653, MC-Hybrid outperformed the current state-of-the-art Li. et al [29] (83.5% accuracy) by over 9.5% improvement in accuracy. Notably, MC-Hybrid auto-extracted features from raw sensor data without any manual feature engineering. The results are presented in Table 5.

### C. MC-HYBRID VS BASELINE MODELS

Our study compared MC-Hybrid with both DL and traditional ML baseline models. The deep learning baseline models auto-extracted features from the raw sensor data. For traditional ML baseline models, we manually engineered gait-related features, and used the mutual information feature selection algorithm to select the top 30 gait features, which were then classified using traditional machine learning baselines. The features listed in Table 7 are the top-ranking features selected for the machine learning baseline models, based on feature importance analysis and prior domain knowledge of gait features that capture alcohol-induced impairments.

Equation (16) expresses the mutual information feature selection algorithm.

(16) $$I(X;Y)=\sum_{x\in 𝒳}\sum_{y\in 𝒴}p(x,\;y)log\left(\frac{p(x,\;y)}{p(x)p(y)}\right)$$

Here, p(x,y) is the joint probability of X and Y,p(x) and p(y) are the marginal probabilities. MC-Hybrid’s performance vs. baselines performance results is shown in Table 6. From the results, it is shown that our novel approach outperformed all baselines by more than 9.0% accuracy, without requiring manual feature engineering.

### D. COMPARISON OF DIFFERENT WINDOW SIZES FORPROPOSED MC-HYBRID MODEL VS. BASELINES

We evaluated the performance of our proposed MC-Hybrid model and other machine learning models for different window sizes. We observed that an increase in window size improved the overall model performance for both MC-Hybrid and baseline ML models. MC-Hybrid model performed the best with a window size of 1024 ( 4.88 seconds) with a 50% overlap. As shown in Table 8, MC-Hybrid outperformed all baseline models for all window sizes evaluated.

### E. COMPARISON OF DIFFERENT ATTENTION MECHANISMS FOR MC-HYBRID MODEL

The performance achievable using different attention mechanisms per channel were compared to determine the best option as a component in MC-Hybrid. We evaluated Multi-Head Attention (MHA) [41], cross [45], channel-wise [46], temporal [47], and self-attention [48] mechanisms. MHA, expressed by (17), captures multiple views for complex features. Cross attention expressed by (18), aligns multi-sensor data. Temporal attention and self-attention expressed in (19) and (21) respectively, focus on predictive timepoints and capture time-step dependencies. Self-attention outperformed other attention mechanisms by **> 2%** improvement in accuracy. Table 9 summarizes the results of different attention mechanisms.

(17) $$MHAc\left(Q_{c},K_{c},V_{c}\right)=Concat\left(\mathrm{head}1,c,\ldots ,{\;\mathrm{head}}_{h,c}\right)W_{c}^{O}$$

$${head}_{i}=Attention\left(QW_{i}^{Q},KW_{i}^{K},VW_{i}^{V}\right)$$

(18) $${\text{Cross-Attention}}_{c}\left(Q_{c},K_{c},V_{c}\right)=\text{Softmax}\left(\frac{Q_{c}K_{c}^{T}}{\sqrt{d_{k}}}\right)V_{c}$$

(19) $$Temporal\;Attention\;\left(\alpha_{t,c}\right)=\frac{exp\left(score\left(h_{t,c},h_{\text{query},c}\right)\right)}{\sum_{t^{\prime }}exp\left(score\left(h_{t^{\prime },c},h_{\text{query},c}\right)\right)}$$

(20) $$Channel\;Attention(X)=\sigma \left(W_{1}\cdot ReLU\left(W_{2}\cdot X\right)\right)$$

(21) $${\text{Self-Attention}}_{c}\left(X_{c}\right)=\;\text{Softmax}\;\left(\frac{X_{c}X_{c}^{T}}{\sqrt{d}}\right)X_{c}$$

### F. DEMONSTRATING GENERALIZATIONS ACROSSSUBJECTS

**FIGURE 13.**
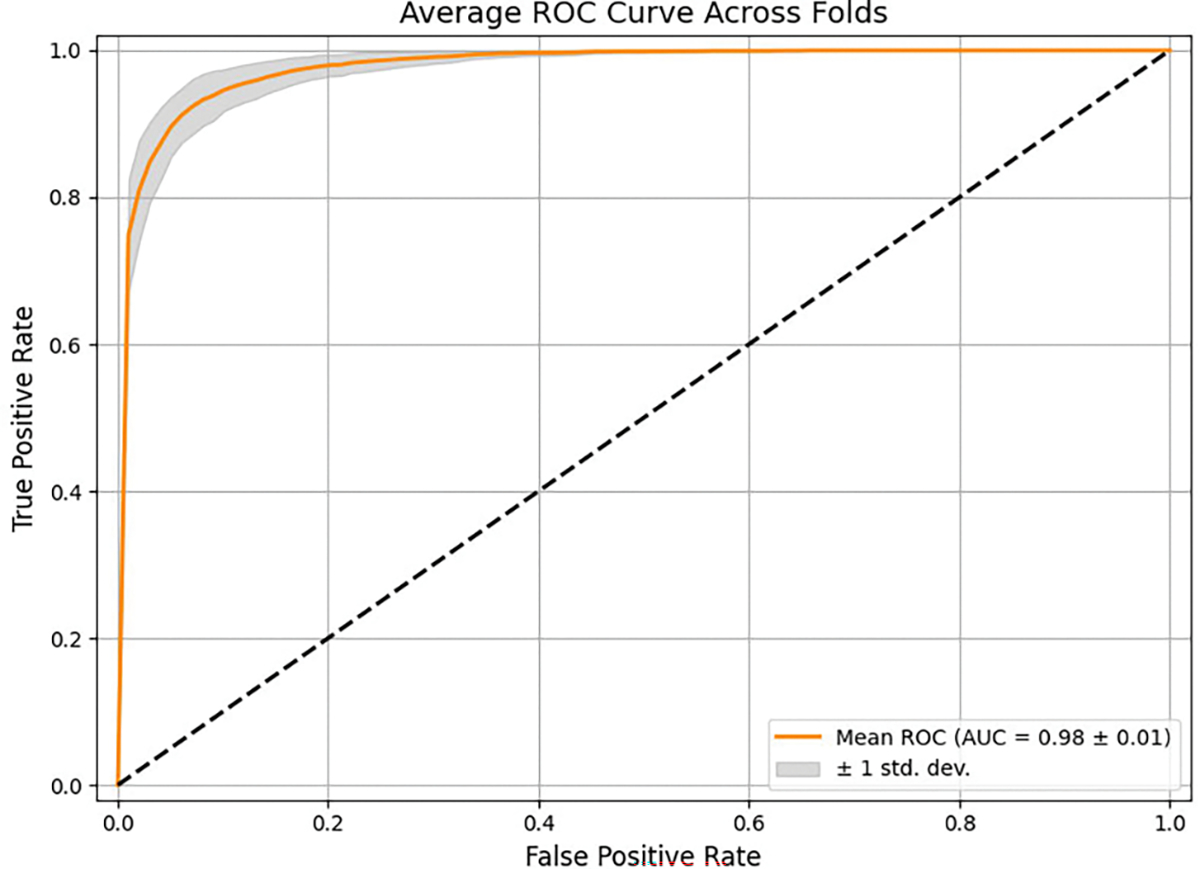
Average Five-fold cross-validation ROC curve.

**FIGURE 14.**
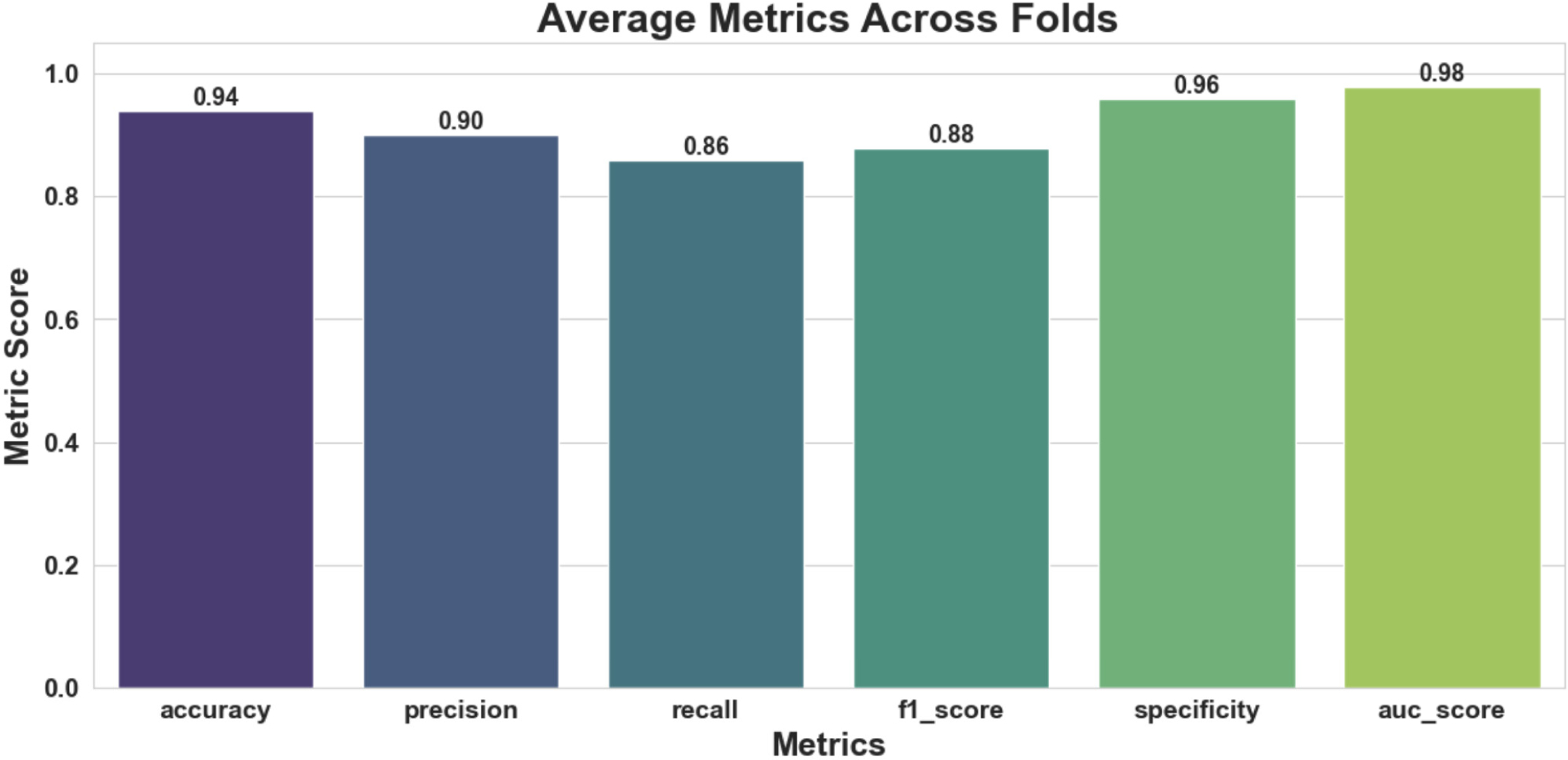
Five-fold cross-validation average metrics bar plot.

Subject-level five-fold cross-validation was conducted to evaluate MC-Hybrid’s ability to generalize across different subjects. Our results are reported in Table 10. Low variance and high AUC scores for each fold show model stability and strong discriminative performance across subjects. Furthermore, consistent F1 scores across folds suggest robust performance despite class imbalance and subject variability. Overall, MC-Hybrid generalizes well to unseen subjects with mean accuracy, AUC score and F1 scores of 0.9374, 0.9807, and 0.8784 respectively. Figures 13 and 14 show the average ROC curve and metrics bar plots across the five folds, highlighting the strong performance of MC-Hybrid.

### G. MODEL INTERPRETABILITY

To confirm that the representations learned by MC-Hybrid are meaningful, we visualized attention weights and performed channel ablation on the test set.

#### 1) ATTENTION WEIGHT ANALYSIS

**FIGURE 15.**
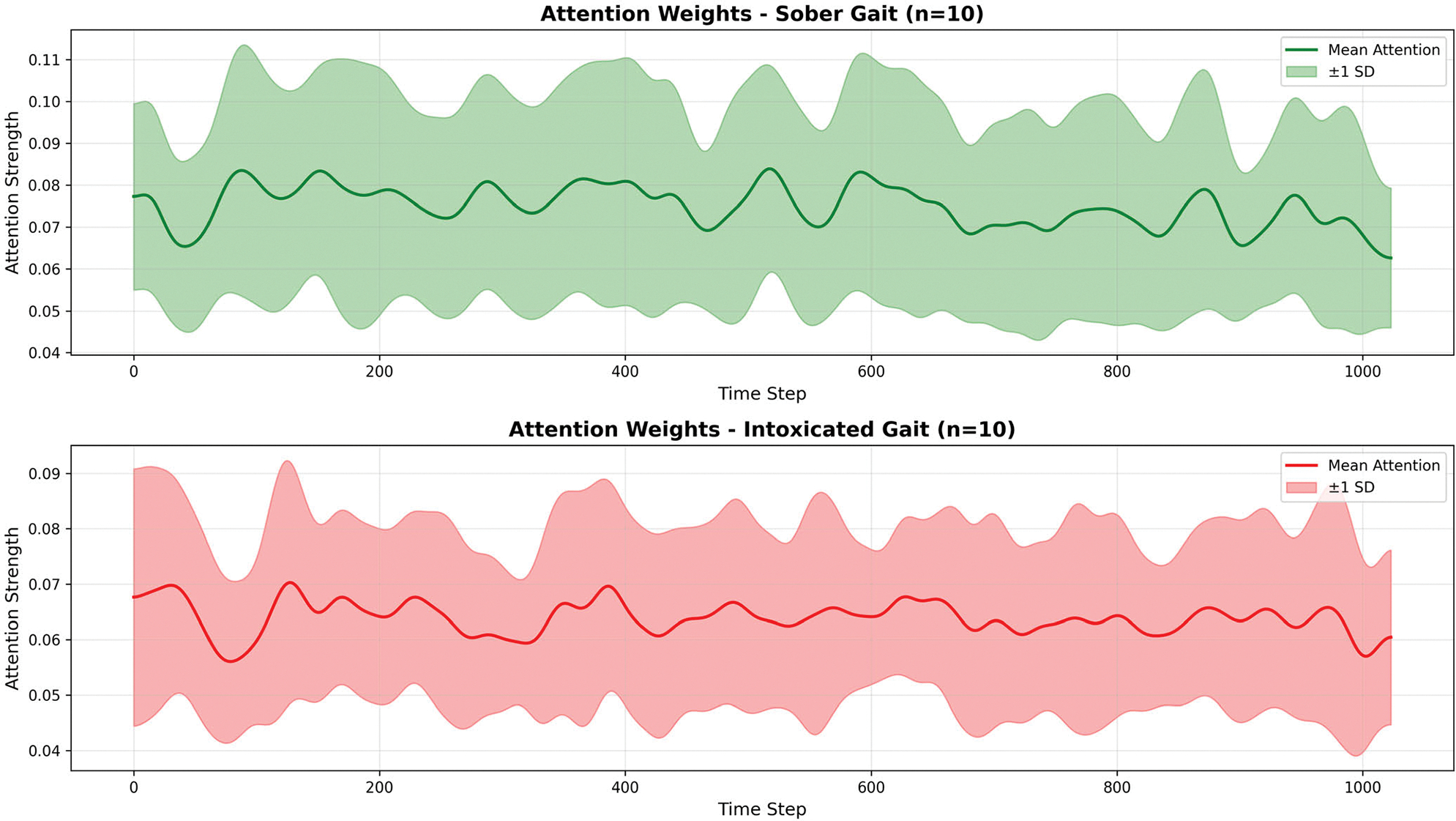
Attention weights distribution for sober and intoxicated samples.

We extracted attention weights from 10 randomly selected samples per class, applying Gaussian smoothing (σ=10) to reveal temporal patterns. Figure 15 shows the mean attention distribution across the 1024-timestep input windows. We observed that sober samples exhibit consistently higher attention weights (mean: 0.074 ± 0.012) compared to intoxicated samples (mean: 0.064 ± 0.009). This 13.5% difference suggests MC-Hybrid learned that intoxicated patterns are more stereotyped and require less distributed attention for classification. In gait analysis, a stereotyped gait refers to a repetitive, rhythmic and non-purposeful walking pattern. In contrast, sober patterns exhibited greater variability requiring more attentive analysis. This is consistent with clinical observations that intoxicated gait exhibits stereotyped ataxic patterns [49]. More attention visualizations are found in the Supplementary figures and tables section in the Appendixes section.

##### a: TEMPORAL DISTRIBUTION

**FIGURE 16.**
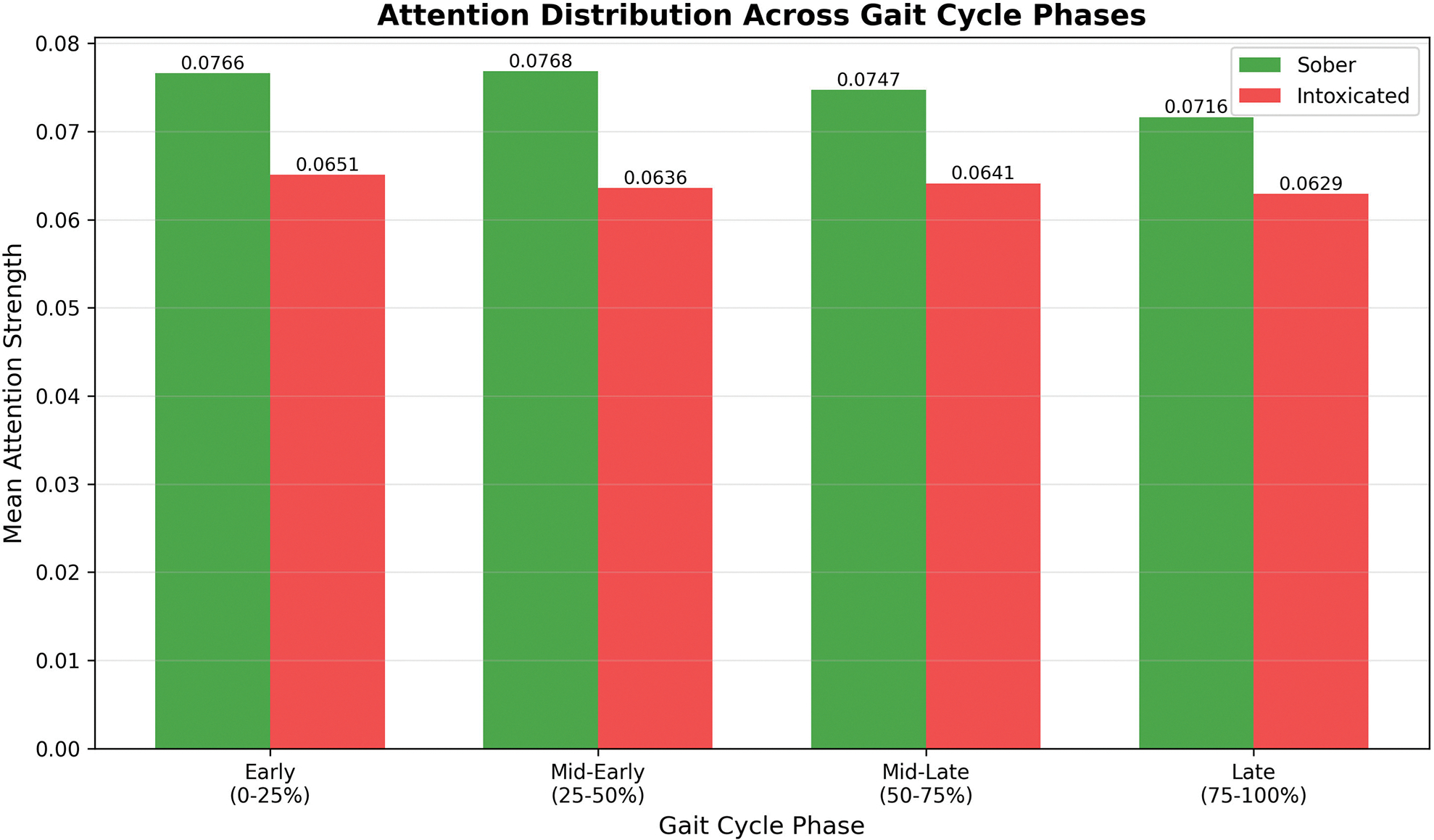
Attention distribution across gait cycle.

Partitioning the gait time series data into quartiles revealed the attention distribution across the gait cycle (as shown in Table 11 and Figure 16). The largest discrimination between intoxicated and sober classes occurs in the 25–50% phase ((mid-phase), indicating this temporal segment contains the most class-discriminative information. In human gait biomechanics, this phase corresponds to mid-stance during which weight transfer occurs and single-limb support requires maximum balance control. Overall, MC-Hybrid learns specific discriminative patterns in gait sub-sequences rather than relying on global statistics.

#### 2) CHANNEL ABLATION STUDY

**FIGURE 17.**
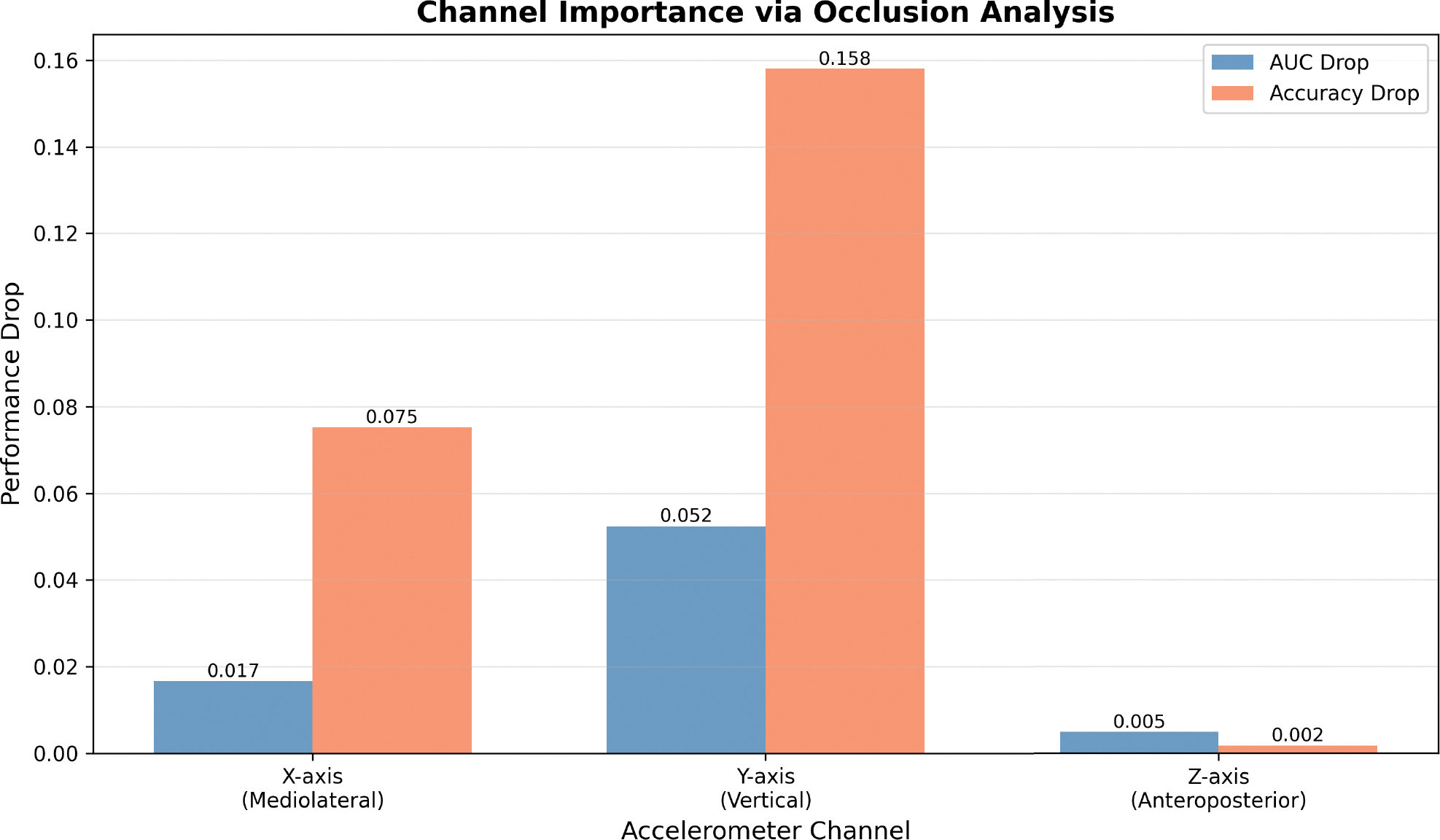
X,Y, Z Channels importance ranking.

We performed channel ablation by systematically masking each input channel and measuring AUC and Accuracy performance degradation. From Figure 17 and Table 12, the Y-axis shows significant importance (15.8% accuracy drop), validating automatic feature selection whereby MC-Hybrid down-weights less informative channels during training. The 79× importance ratio (Y:Z) confirms hierarchical learning. This aligns with clinical observations that alcohol primarily affects postural stability and balance [50], which field sobriety tests specifically assess [51]. On the other hand, Z-axis had minimal impact (0.5% AUC drop) when removed. This makes sense as the smartphone utilized for data collection was strapped to the torso of participants with its screen front facing. Intoxication is well known to manifest as a side-to-side swaying of the torso, which corresponds to the data collection phone’s X-Y plane. As such, removal of the Z axis had minimal impact on the model’s accuracy in detecting intoxication. Even though the combined channels outperformed single channels, our findings show that single Y-axis deployment could retain ~95% performance with 67% data reduction. Real-time and low computation resource systems could process only Y-axis data for computational efficiency.

### H. ABLATION STUDY

We performed an ablation study to determine the contributions of each component of our new MC-Hybrid model. In each experiment, one component of the MC-Hybrid model was removed and its performance re-evaluated using various metrics. As shown in Table 13, MC-Hybrid outperforms all the ablated versions across all metrics, achieving an accuracy of 93% and AUC score of 0.98. Removing the self-attention layer resulted in a drop in model performance with F1 score dropping from 0.8653 to 0.8238, and AUC score dropping from 0.98 to 0.97. This highlights the critical role of attention in capturing salient alcohol intoxicated gait features. Removing the CNN layer significantly reduced MC-Hybrid’s accuracy to 88.48% and F1 score to 0.79, indicating its importance for feature extraction from the input data. Although removal of bidirectionality slightly affected AUC and accuracy, the impact on performance was less pronounced than for other components, suggesting that unidirectional mechanisms could still retain sufficient temporal information for alcohol intoxication detection. These findings reveal that each component of MC-Hybrid (attention, bidirectionality, CNN, LSTM) contributes non-trivially to its performance and that combining them results in the highest performance across all metrics.

## V. DISCUSSION

**Proposed MC-Hybrid outperforms all previous approaches** by **> 9.5%.** MC-Hybrid surpasses the DL model proposed by Li et al. [29], the state-of-the-art study that utilized the same dataset. This confirms the outperformance of MC-Hybrid on real-world alcohol gait data. It is also instructive to note that comparisons with the work of Aiello and Agu [26] are not directly equivalent as their work simulated impairment from drunk buster goggles instead of real alcohol consumption. MC-Hybrid also outperformed traditional machine learning and deep learning baselines by > 8.04% in accuracy for smartphone accelerometer-based time series intoxicated gait analyses task. This demonstrates that our proposed multi-component MC-Hybrid framework effectively captured intricate temporal and spatial dependencies and suggests that the unique combination of such hybrid components can improve performance in complex time series problems.

**Larger window sizes improved model performance:** and highlighted the importance of choosing the optimal window size and its impact on model performance. After experimenting with different window sizes (256, 512 and 1024), we found that increasing window size generally increased performance with a window size of 1024 ( 4.88 seconds) yielding optimal performance.

**Self-attention outperformed other attention mechanisms** by 2%, and improved overall model performance. This improvement was because self-attention uses context-sensitive mechanism, enabling MC-Hybrid to focus on the most predictive parts of the gait data and important gait patterns, while ignoring noise.

**Model Interpretability:** Our attention and ablation analyses reveal that the MC-Hybrid model learns a hierarchical feature representation with clear interpretability. The vertical channel (Y-axis) demonstrates overwhelming importance (15.8% accuracy drop when masked), indicating the model automatically prioritizes the most discriminative signal dimension. The attention mechanism revealed differentiated patterns between classes, with sober samples requiring 13.5% higher attention due to greater pattern variability. Temporal analysis reveals that the 25–50% phase contains maximum discriminative information (17.3% attention difference), suggesting the model focuses on specific gait time series sub-sequences rather than global features. These findings demonstrate that the MC-Hybrid model learns interpretable, task-relevant representations aligned with domain knowledge, increasing confidence in its generalization and deployment potential. Future work could explore attention-guided data augmentation or pruning less important temporal segments to further increase computational efficiency.

**IRB-approved actual alcohol dataset improves result.** While hybrid CNN-RNN-attention models are established for time-series tasks, our MC-Hybrid (with parallel multi-channel design) innovates by applying them to triaxial gait data, further improving performance gains. However, we acknowledge that performance gains may also be amplified by our comprehensive pre-processing pipeline and large, high-quality dataset collected in an IRB-approved controlled experiment in which participants consumed real alcohol. This dataset provides a cleaner, higher fidelity, more representative and ecologically valid gait signals than simulated data.

**Addressing class imbalance through random oversampling:** To address class imbalance, we evaluated random oversampling, SMOTE, and undersampling class imbalance mitigation approaches. SMOTE produced synthetic gait sequences that often lacked physiological plausibility, while random undersampling discarded valuable sober data and reduced variability. In summary, both approaches degraded model performance. Despite its simplicity, random oversampling preserved the original signal distribution and yielded the most stable results. We plan to investigate more adaptive approaches such as ADASYN in future work.

## VI. LIMITATIONS AND AREAS FOR IMPROVEMENT

### A. MISCLASSIFICATION OF CERTAIN BAC LEVELS

**FIGURE 18.**
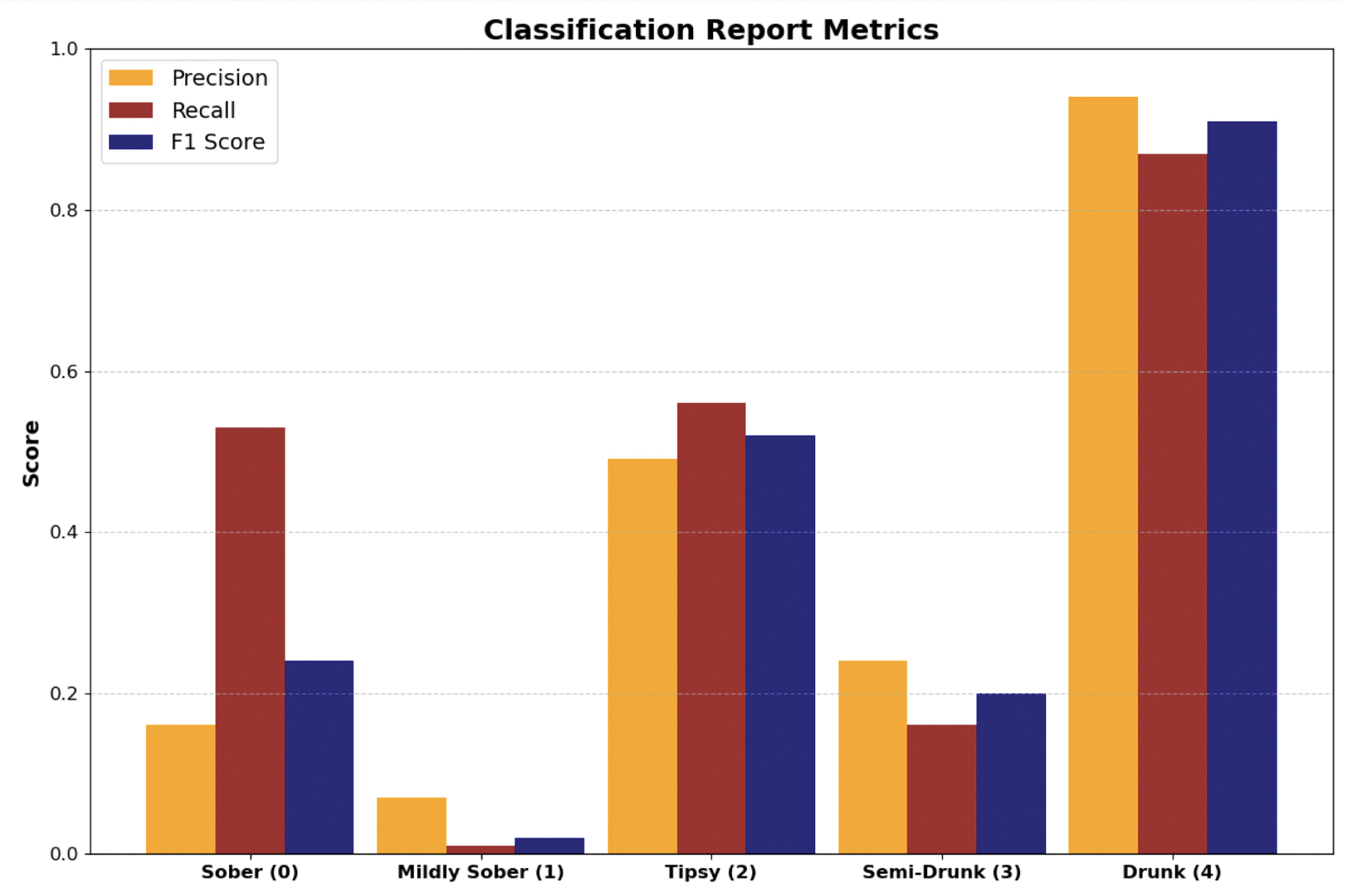
MC-Hybrid classification Metrics.

While MC-Hybrid performs well as a binary classification model, demonstrating its utility for practical DUI detection, it struggles to classify some early-stage intoxication BAC levels. Considering five classes of BAC levels - sober (0), mildly sober ([0.0 – 0.030)), tipsy ([0.03 – 0.06)), semi-drunk ([0.06 – 0.08)) and drunk ([0.08 - )), we evaluated MC-Hybrid’s classification on each of the class. The result shown in Fig. 18 showed that MC-Hybrid achieved the highest classification performance on class 4 (drunk), moderate classification performance in class 2 (tipsy) and class 0 (sober), while struggling with classification of BAC samples in class 3 (semi-drunk) and class 1 (mildly sober). These findings highlight a common challenge in distinguishing borderline BAC levels in real life due to overlapping gait patterns and subtle impairments in these ranges.

### B. NON-TRIVIAL FALSE POSITIVE RATE

Although our proposed MC-Hybrid achieved a high recall of 0.9192, it also had a non-trivial false positive rate (0.8174 precision), which should be improved. Such a false positive rate implies that MC-Hybrid classifies intoxicated samples correctly, but has a slight tendency to classify sober samples as intoxicated, a false alarm in real-world usage. In real-world applications such as vehicle immobilization, false alarms could become a nuisance with social and usability implications such as immobilizing a sober subject’s vehicle that would cause embarassment and inconvenience, and potentially erode user trust in the system. A practical solution to mitigate this issue in the real world is to enforce temporal consistency, requiring multiple consecutive windows (e.g., 3–5) to be classified as “intoxicated” before triggering an intervention. This leverages gait continuity and reduces false alarms from brief fluctuations. A separate additional temporal classifier could also be trained to enforce the number of consecutive instances that need to be classified as intoxicated, subject to specific operational needs and local laws. Additionally, applying a confidence threshold (e.g., ≥ 95%) before taking action can balance sensitivity and specificity–though higher thresholds may increase false negatives. Detailed experimentation will be required to select the optimal threshold, which should reflect acceptable risk tolerance and costs of erroneous inference. Future work should empirically validate these strategies on held-out data or via user studies to confirm their effectiveness in reducing false positives without compromising safety or usability.

### C. MITIGATING CONFOUNDING FACTORS

We previously acknowledged confounding factors such as stress, fatigue, and minor injuries, which may alter gait patterns in ways similar to alcohol intoxication. A critical next step is to experimentally design studies to develop methods to isolate and differentiate these effects. A promising direction is to collect controlled gait data capturing other non-intoxication impairments. For instance, participants could be recorded: after strenuous activity (e.g., treadmill running) to simulate fatigue, with mild physical constraints (e.g., knee braces or ankle weights), and during validated stress-inducing tasks to capture stress-altered gait. Comparing these datasets with alcohol-impaired gait and performing joint modeling via methods such as multi-task learning could reveal distinctive patterns or enable multi-class classification to distinguish impairment types. Alternatively, multi-modal sensing (e.g., combining gait with speech, heart rate, or facial cues) could help disambiguate impairment causes. Such studies could enhance model robustness and extend it beyond binary detection, toward impairment attribution.

### D. CLASS IMBALANCE

Our dataset had a limited number of alcohol intoxicated samples. Table 14 shows the imbalance in our dataset. Training the model on an imbalanced dataset would have yielded a model that was slightly biased to the more abundant sober class. To mitigate this, we oversampled the number of intoxicated samples in the train set. Hence, our model achieved a strong F1 score of 0.8653, indicating balance between recall and precision. Although random oversampling was the most effective class imbalance mitigation approach in our experiments, it works by duplicating samples, which may increase the risk of overfitting. SMOTE and undersampling yielded poorer results, and ADASYN remains an avenue for combining gait data balancing together with domain-specific augmentations to possibly improve MC-Hybrid’s generalization to the minority class in future work.

### E. LACK OF PUBLICLY AVAILABLE ALCOHOL DATASET

Experiments evaluating MC-Hybrid on additional publicly available datasets would have validated our results and verified that MC-Hybrid generalizes well to other datasets. However, after an extensive search, we were unable to find publicly available alcohol intoxication gait datasets.

### F. SUBJECT-LEVEL Z-SCORE NORMALIZATION: JUSTIFICATION AND TRADE-OFFS

We applied subject-level Z-score normalization to reduce inter-subject variability in gait signals arising from natural differences in stride, pace, and motion range. This standardization improved model stability by aligning input distributions across subjects. However, it may also attenuate informative inter-subject variance—if alcohol increases gait variability (e.g., inconsistent stride length or lateral sway), normalizing by each subject’s own standard deviation could mask these effects and reduce the sensitivity of intoxication detection. Alternatively, global normalization—standardizing across all subjects—preserves inter-subject differences but risks overfitting to subject-specific biases, reducing generalization. We therefore prioritized subject-level normalization to capture within-subject deviations from a sober baseline. Future work could investigate hybrid normalization or architectures that jointly model normalized and raw features, leveraging both within- and between-subject dynamics.

### G. DEVICE VARIABILITY

Differences in smartphone hardware from different manufacturers may cause variability of sensor outputs for the same inputs, which may impact the accuracy of ML models. In real world usage, in addition to device-specific variability, environmental noise and phone orientation can all impact data quality and the performance of ML intoxication detection models.

### H. FIXED DEVICE ORIENTATION

To ensure consistent placement across participants, the smartphones used for data collection in our study were secured in a standardized waist holster strapped to the participants’ waist. This minimized noise but also created a best-case scenario for classification performance. In real-world deployments, phones may be carried loosely in pockets, bags, or held in the hand, causing significantly higher signal variability. Thus, our reported 93% accuracy should be interpreted as an upper bound, with performance in-the-wild expected to be lower.

## VII. CONCLUSION

Driving under the influence of alcohol remains a significant cause of fatalities worldwide, emphasizing the need for effective intervention methods. While accurate, breathalyzers require carrying additional hardware, and are not suitable for real-time continuous monitoring. This highlights the necessity for a non-invasive, reliable solution. This paper proposed the MC-Hybrid DUI detection framework that learns predictive patterns from raw gait data without requiring manual feature engineering. The MC-Hybrid pipeline consists of a set of innovative pre-processing steps including stratified subject-level split, normalization, outlier removal, noise filtering, window segmentation. To mitigate class imbalance and prevent bias towards the more abundant sober data samples, the train set was randomly oversampled with minority intoxicated gait samples to match the number of sober gait samples in the train set. MC-Hybrid outperforms previous state-of-the-art methods and established baselines, achieving an impressive accuracy of 93%. It offers a robust and non-invasive alternative for detecting alcohol impairment and potentially saving lives. Its 1D-CNN layer effectively performed low-level short-term gait feature extraction, detecting gait abnormalities associated with alcohol impairment. The attention layer emphasized critical temporal patterns, focusing on balance indicators in gait that could change with alcohol intoxication, and the Bi-LSTM layer modeled long-term sequences in our gait data and highlighted step irregularities. The pooling layer played a key role in detecting key movement attributes, while filtering out noise from the extracted features. Finally, the dense layer refined the features and improved prediction.

## FUTURE WORK

Future work will enhance MC-Hybrid’s performance and practical applicability in the real world. While our study was conducted in a controlled indoor environment, real-world conditions such as uneven terrain, varying lighting, phone displacements, or crowd movement may introduce noise to smartphone-sensor-captured gait signals. A future research direction will focus on evaluating the MC-Hybrid model in uncontrolled environments to investigate its generalizability. Noise mitigation will also be explored using approaches such as domain adaptation techniques. Techniques such as Domain-Adversarial Neural Networks (DANN) can reduce shifts in distribution between controlled and real-world scenarios by learning domain-invariant features during training, enhancing robustness in real-world deployment scenarios. To address the scarcity of public alcohol datasets, we plan to apply cross-domain transfer learning, pre-training on publicly available human activity datasets before fine-tuning on our alcohol gait dataset. We will also explore synthetic data generation using GANs or other generative models to mitigate class imbalance. Regarding the fixed phone placement on the waist, the lumbar region (torso and including the waist) are the most stable locations on the human body. Consequently, many gait studies attach sensor devices to the lumbar region especially as a first investigation. Moreover, most phone owners also carry their phones on their lumbar region. We plan to collect data and investigate the impact of carrying the smartphone on other locations in our alcohol gait studies. Methodologically, we aim to improve on precision and F1 scores achieved in this work by implementing precision-sensitive loss functions such as focal loss and precision-recall AUC optimization, or via multimodal fusion with complementary sensors (GPS, or contextual behavioral data). Additionally, although a parallel multi-channel design was selected in this paper to preserve axis-specific temporal features, future research will systematically benchmark simpler concatenated-channel variants in order to quantify the performance–complexity trade-off. Other directions include analyzing and modeling cross-axis accelerometer relationships, converting 1D signals into 2D representations to leverage image-based DL architectures, and extending target labels beyond classification to regression of nominal BAC values and anomaly detection. Finally, we also plan to explore the effectiveness of our MC-Hybrid framework in detecting gait impairment caused by other sources such as the consumption of other substances (e.g. marijuana) and fatigue.

## Figures and Tables

**FIGURE 6.**
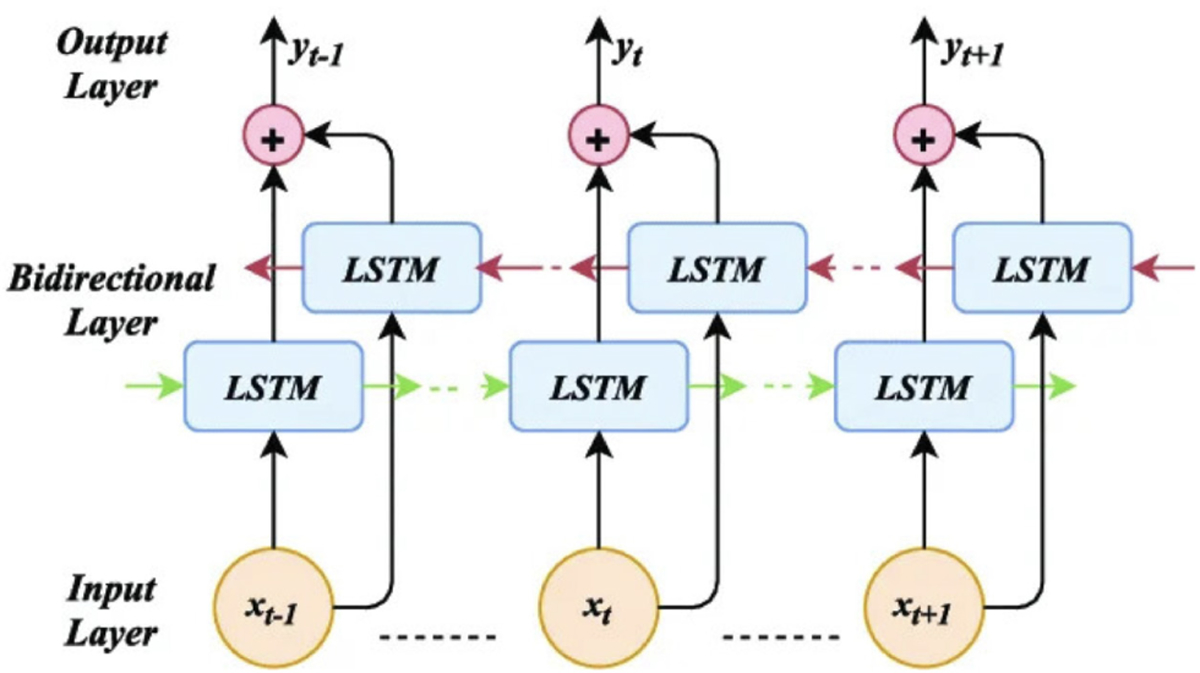
Bi-LSTM:Deep Learning Baseline.

**FIGURE 19.**
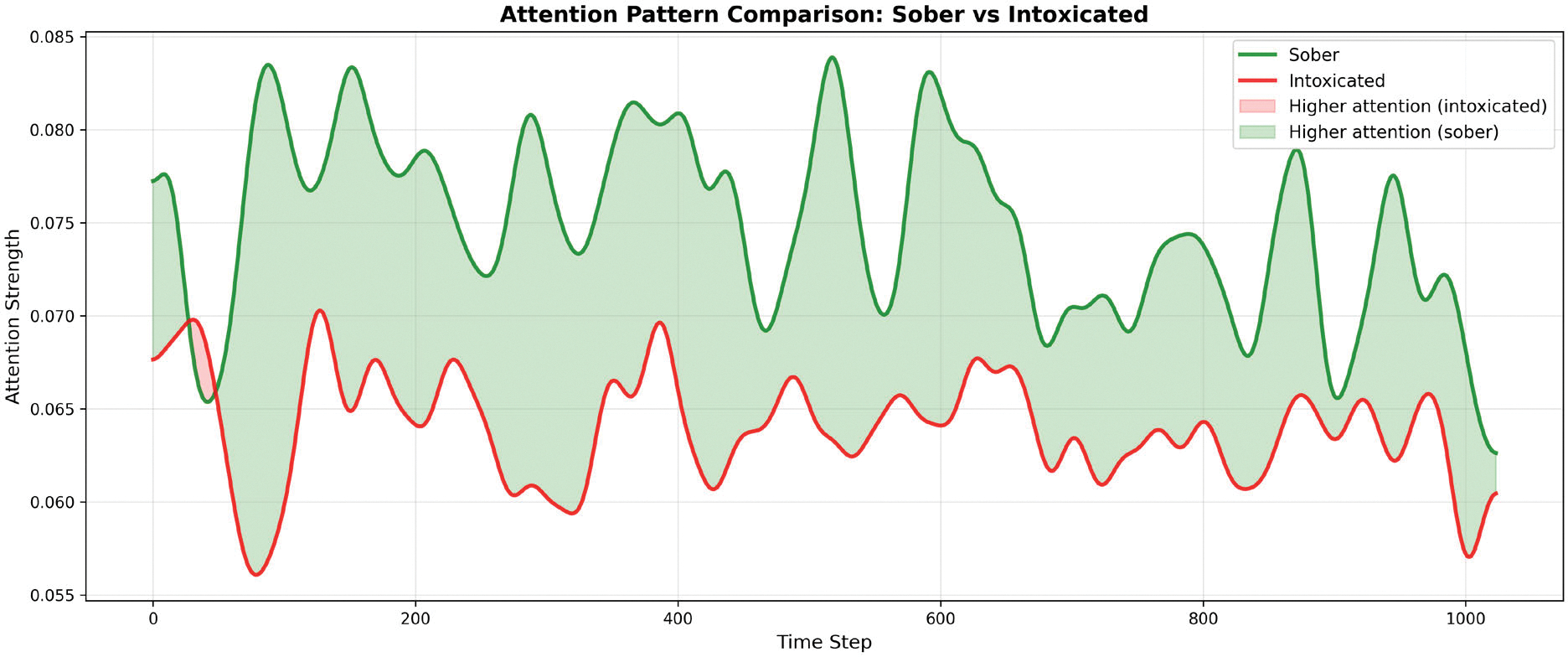
FIGURE S1. Direct overlay comparison of mean attention weights for sober(green) and intoxicated (red) gait patterns across the 1024-timestepinput window (n=10 samples per class). Green shading indicates timesegments where sober attention exceeds intoxicated attention, demonstratingconsistent higher attention requirements throughout the sequence.

**FIGURE 20.**
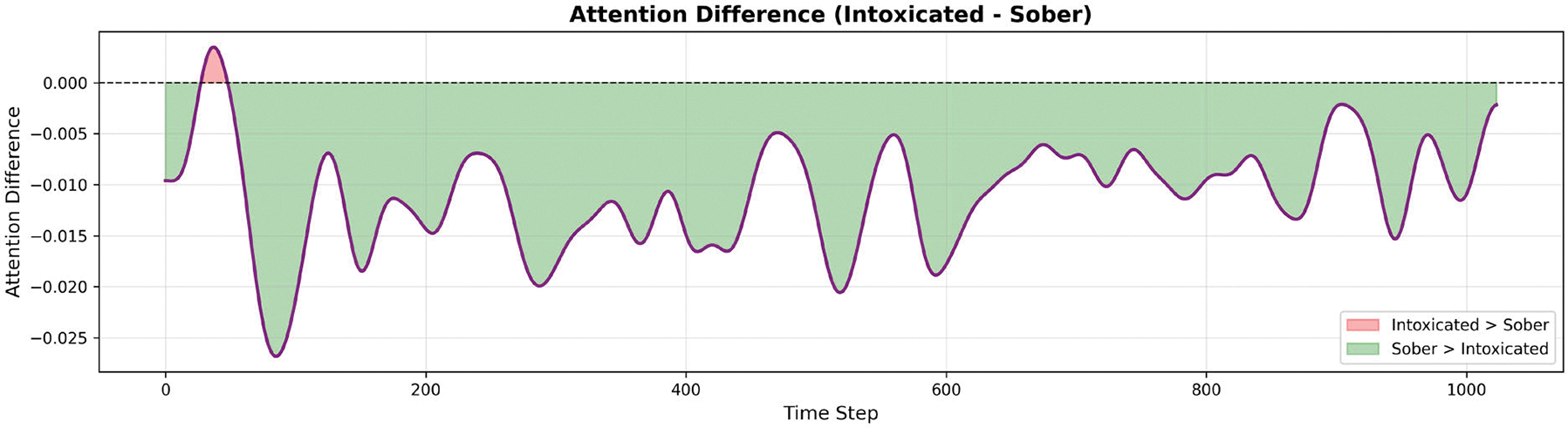
FIGURE S2. Attention difference computed as (Intoxicated – Sober) meanattention weights. Consistently negative values (range: −0.027 to −0.002)indicate sober samples require higher attention at all time steps, withoscillatory variation reflecting gait cycle periodicity.

**TABLE 1.** Previous smartphone classification studies on alcohol intoxication detection from gait.

Method type	Method	Accuracy	Target classes	Source
**Traditional ML**	RF (SNR, Cadence, Skewness, Kurtosis)	70%	Sober (0.2 drinks), tipsy (3–6 drinks), drunk (>6 drinks)	Arnold *et al.* [23]
	J48 classifier (Sway Area, Sway Volume, Kurt, Skew, Gait Velocity)	89.45%	[0.00–0.08), [0.08–0.15), [0.15–0.25), [0.25+)	C. Aiello and Agu [26]
**Deep Learning (state of the art)**	**Bi-CNN (Raw sensor data)**	**83.5%**	**Sober, drunk**	**Li** *et al.* **[29]**

**TABLE 2.** Previous studies in smartphone regression approach to alcohol intoxication detection from gait.

Method Type	Method	RMSE	Source
**Deep Learning**	MLP	0.0226	Gharani P. *et al.* [30]
	Bi-LSTM	0.0167	Li *et al.* [31]
	CNN	0.0168	Li *et al.* [31]

**TABLE 3.** Dataset split showing the distribution of samples across train, validation, test, and overall splits.

Dataset Split (Train, Validation, Test)				Overall
Split Type	# Samples (Train 70%)	# Samples (Validation 10%)	# Samples (Test 20%)	# Samples
**Sober (0)**	9,710,973	1,434,322	3,048,382	14,193,677
**Intoxicated (1)**	3,291,129	526,825	947,092	4,765,046
**Split Total**	13,002,102	1,961,147	3,995,474	18,958,723

**TABLE 4.** Optimal hyperparameters for MC-Hybrid.

Hyperparameter	Value
Optimizer	Adam
Learning Rate	0.00024227083179696293
Window Size	1024
Number of Channels	3
Number of Classes	1
Filters (1st Conv1D)	117
Kernel Size (1st Conv1D)	6
Filters (2nd Conv1D)	175
Kernel Size (2nd Conv1D)	6
Activation (Conv1D Layers)	ReLU
Padding (Conv1D Layers)	Same
LSTM Units	85
LSTM Return Sequences	True
Pooling Layer	GlobalAveragePooling1D
Dense Layer 1 Units	129
Dense Layer 2 Units	188
Output Layer Units	1
Output Layer Activation	Sigmoid
Dropout Rate	0.696581562334145
Loss Function	Binary Crossentropy
Metrics	Accuracy, AUC

**TABLE 5.** Results of our approach vs prior works.

Method	Accuracy	F1-score	Source
Bi-linear CNN	83.5%	—	Li *et al.* [29]
J48 classifier	89.45%	—	Aiello and Agu [26]
Random Forest	70%	—	Arnold *et al.* [23]
**Multichannel hybrid**	**93%**	**0.8653**	**Our approach (MC-Hybrid)**

**TABLE 6.** Performance metrics for our approach vs. baseline models.

Model	Accuracy	AUC	F1_score	Precision	Recall	Specificity

**Proposed Model**						

**MC-Hybrid**	**0.93**	**0.98**	**0.8653**	**0.8172**	**0.9192**	**0.9285**

**Deep Learning Baselines**						

1D-CNN	0.8609	0.9545	0.7707	0.6708	0.9057	0.8452
Bi-LSTM	0.84	0.8380	0.6739	0.6949	0.6541	0.90
LSTM-GRU	0.7559	0.8069	0.6133	0.5190	0.7495	0.7559
LSTM	0.8040	0.7423	0.5698	0.6575	0.5027	0.9088
SLP	0.7843	0.8614	0.6584	0.5570	0.8050	0.7772

**Traditional Machine Learning Baselines**						

SVM	0.8428	0.8825	0.7079	0.6804	0.7376	0.8794
Naïve-Bayes	0.6342	0.6495	0.4688	0.3750	0.6250	0.6374
RF	0.8424	0.8961	0.5920	0.8925	0.4429	0.9814
Decision-Tree	0.7061	0.6170	0.4327	0.4313	0.4327	0.8014
KNN	0.6719	0.6398	0.4084	0.3822	0.4386	0.7531
LOG-REG	0.7213	0.7938	0.5826	0.4750	0.7532	0.7102

**TABLE 7.** Top-ranking features used for machine learning baselines.

Feature Name	Mathematical Representation (Axes)
**Maximum Value**	max(*X*), max(*Y*), max(*Z*)
**Maximum-Minimum Difference**	max(*X*) – min(*X*), max(*Y*) – min(*Y*), max(*Z*) – min(*Z*)
**Minimum Value**	min(*X*), min(*Y*), min(*Z*)
**Mean Value**	$\frac{1}{n}\sum_{i=1}^{n}Y_{i}$
**Standard Deviation**	$\sqrt{\frac{1}{n}\sum_{i=1}^{n}\;{\left(X_{i}-\mu_{X}\right)}^{2}},\sqrt{\frac{1}{n}\sum_{i=1}^{n}\;{\left(Z_{i}-\mu_{Z}\right)}^{2}}$
**Band Power**	$\int_{f_{1}}^{f_{2}}\;|X(f){|}^{2}df,\int_{f_{1}}^{f_{2}}\;|Z(f){|}^{2}df$
**Root Mean Square (RMS)**	$\sqrt{\frac{1}{n}\sum_{i=1}^{n}\;X_{i}^{2}},\sqrt{\frac{1}{n}\sum_{i=1}^{n}\;Z_{i}^{2}}$
**Energy in Frequency Domain**	$\sum |X(f){|}^{2},\sum |Z(f){|}^{2}$
**Spectral Entropy**	$\begin{array}{r} -\sum P_{X}(f)logP_{X}(f)\\ \;-\sum P_{Y}(f)logP_{Y}(f)\\ \;-\sum P_{Z}(f)logP_{Z}(f) \end{array}$
**Spectral Bandwidth**	$\sqrt{\sum {\left(f-\mu_{f}\right)}^{2}P_{X}(f)}$
**Absolute Average Deviation (AAD)**	$\frac{1}{n}\sum_{i=1}^{n}\;\left|X_{i}-\mu_{X}\right|,\frac{1}{n}\sum_{i=1}^{n}\;\left|Z_{i}-\mu_{Z}\right|$
**Kurtosis**	$\frac{\frac{1}{n}\sum_{i=1}^{n}\;{\left(X_{i}-\mu_{X}\right)}^{4}}{\sigma_{X}^{4}}$
**Skewness**	$\frac{\frac{1}{n}\sum_{i=1}^{n}{\left(X_{i}-\mu_{X}\right)}^{3}}{\sigma_{X}^{3}}$
**Sway Volume**	$\text{Volume}=\int_{t_{0}}^{t_{1}}|\vec{S}(t)|dt$
**Sway in XZ Plane**	${\text{Sway}}_{XZ}=\sqrt{X^{2}+Z^{2}}$
**Signal Magnitude Area (SMA)**	$\text{SMA}=\frac{1}{n}\sum_{i=1}^{n}\;\left(\left|X_{i}\right|+\left|Y_{i}\right|+\left|Z_{i}\right|\right)$
**Average Resultant Acceleration in Frequency Domain**	$\frac{1}{n}\sum_{i=1}^{n}\;\sqrt{X_{f}^{2}+Y_{f}^{2}+Z_{f}^{2}}$

**TABLE 8.** Comparing the impact of different window sizes.

Window size	Model	Accuracy	AUC Score	Precision	Recall	F1 Score	Specificity
256	SVM	0.7803	0.8713	0.5519	0.7877	0.649	0.7778
	Random Forest	0.8234	0.899	0.63	0.7217	0.6732	0.8588
	KNN	0.7746	0.8437	0.5477	0.7214	0.6227	0.8865
	Naive Bayes	0.7803	0.8069	0.5718	0.5897	0.5806	0.8466
	Decision Tree	0.7541	0.7363	0.5171	0.6996	0.5947	0.7731
	Gradient Boosting	0.7989	0.8973	0.5818	0.7822	0.6672	0.8047
	XGBoost	0.8067	0.8996	0.5977	0.7649	0.6711	0.8212
	**MC Hybrid**	**0.8761**	**0.9536**	**0.7132**	**0.8207**	**0.7632**	**0.8939**
512	SVM	0.8203	0.915	0.6096	0.8429	0.7075	0.8124
	Random Forest	0.8611	0.9409	0.694	0.8257	0.7541	0.8735
	KNN	0.8249	0.8975	0.6264	0.7957	0.701	0.8351
	Naive Bayes	0.8347	0.8881	0.6779	0.6845	0.6811	0.887
	Decision Tree	0.8014	0.7921	0.5873	0.7731	0.6675	0.8112
	Gradient Boosting	0.8472	0.9411	0.6583	0.8472	0.7409	0.8471
	XGBoost	0.8551	0.941	0.6809	0.8246	0.7459	0.8657
	**MC Hybrid**	**0.8905**	**0.9741**	**0.7188**	**0.904**	**0.8009**	**0.8862**
1024	SVM	0.8792	0.9575	0.7072	0.9084	0.7953	0.8691
	Random Forest	0.9115	0.9769	0.7832	0.9089	0.8414	0.9124
	KNN	0.8865	0.9468	0.7334	0.8804	0.8002	0.8886
	Naive Bayes	0.8773	0.9371	0.7632	0.7608	0.762	0.9178
	Decision Tree	0.8602	0.8589	0.6829	0.8561	0.7597	0.8616
	Gradient Boosting	0.9093	0.9769	0.7712	0.9224	0.84	0.9047
	XGBoost	0.9107	0.9782	0.7808	0.9095	0.8402	0.9111
	**MC-Hybrid**	**0.93**	**0.98**	**0.8174**	**0.9192**	**0.8653**	**0.9285**

**TABLE 9.** Performance of different attention mechanisms.

Attention Type	Accuracy	AUC Score	Precision	Recall	F1 Score	Specificity

**Self-attention**	**0.93**	**0.98**	**0.8174**	**0.9192**	**0.8653**	**0.9285**
MHA	0.89	0.98	0.7224	0.9537	0.8221	0.8724
CROSS	0.91	0.98	0.7739	0.9370	0.8477	0.9047
Temporal	0.82	0.8775	0.6427	0.6927	0.6691	0.8649
Channel-Attention	0.8948	0.97	0.7334	0.9310	0.8205	0.8822

**TABLE 10.** Performance metrics across cross-validation folds.

Fold	Accuracy	AUC Score	F1 Score	Precision	Recall	Specificity

Fold 1	0.9553	0.9897	0.9111	0.9148	0.9074	0.9715
Fold 2	0.9258	0.9738	0.8738	0.8734	0.8742	0.9473
Fold 3	0.9412	0.9866	0.8819	0.9779	0.8031	0.9932
Fold 4	0.9440	0.9872	0.8950	0.8777	0.9129	0.9550
Fold 5	0.9208	0.9661	0.8303	0.8408	0.8202	0.9519

**Average**	**0.9374**	**0.9807**	**0.8784**	**0.8969**	**0.8636**	**0.9638**

**TABLE 11.** Temporal attention distribution.

Phase	Sober	Intox	Δ	Δ%

0–25%	0.0766	0.0651	−0.0115	−15.0
25–50%	0.0768	0.0636	−0.0133	−17.3
50–75%	0.0747	0.0641	−0.0106	−14.2
75–100%	0.0716	0.0629	−0.0087	−12.1

**TABLE 12.** Channel ablation results.

Channel	Axis	ΔAUC	ΔAcc	Relative

Y	Vertical	0.052	0.158	1.00
X	Lateral	0.017	0.075	0.47
Z	Longitudinal	0.005	0.002	0.01

**TABLE 13.** Ablation study result.

Method	Accuracy	AUC Score	Precision	Recall	F1 Score	Specificity
Remove Attention	0.8995	0.9705	0.7529	0.9095	0.8238	0.8961
Remove Bidirectional	0.9200	0.9700	0.8000	0.9203	0.8572	0.9210
Remove CNN	0.8848	0.9600	0.7266	0.8879	0.7992	0.8837
Remove LSTM	0.9000	0.9600	0.7719	0.8863	0.8252	0.9088
**MC-Hybrid**	**0.9300**	**0.9800**	**0.8172**	**0.9192**	**0.8653**	**0.9285**

**TABLE 14.** Distribution of classes in the dataset.

Class	Count	Percentage
Sober	14,193,677	74.87%
Intoxicated	4,765,046	25.13%
**Total**	18,958,723	100%

**TABLE 15.** Confusion matrix skeleton.

	Predicted Negative	Predicted Positive
**Actual Negative**	True Negative (TN)	False Positive (FP)
**Actual Positive**	False Negative (FN)	True Positive (TP)

## APPENDIXES

This section provides supplementary details, including extended methodologies, additional figures, and key equations, to support the findings presented in the main text.

### A. DATASET

Detailed information regarding the dataset collection and experimental procedures is provided below.

#### 1) DATA ACQUISITION PROCESS

A labeled dataset was created by collecting alcohol-intoxicated gait data from 121 participants in a controlled NIH-funded study at Butler Hospital. With an IRB reference number 1612–001, the study was conducted under IRB-approved conditions, supervised by alcohol-controlled-experiment experts. Ethical compliance was ensured, with the study designed in collaboration with alcohol research specialists from Butler Hospital, Rhode Island [29]. The study followed a structured alcohol consumption protocol (Fig. 3). Participants consumed alcohol in controlled doses, underwent breathalyzer tests at regular intervals, and performed repeated walking tasks. A smartphone running a data gathering app captured accelerometer and gyroscope data during each walk session, while BAC was captured before each walking session using a breathalyzer. To minimize variability caused by smartphone placements, all participants carried the device in a standardized waistpack/holster. This approach effectively controlled the potential confounding effects of device placement during data collection. While this controlled setup may not capture all real-world placement scenarios, it provided a clean baseline for initial studying gait-based alcohol detection.

a: ALCOHOL PROTOCOL

#### 2) PARTICIPANT RECRUITMENT AND SCREENING

##### Recruitment process

Participants (ages 21–65) were recruited via online ads and brochures, followed by phone screenings. Eligibility required alcohol consumption at least once in the past month and past consumption exceeding gender-specific thresholds (six drinks per occasion for men, four for women). Additional criteria included drinking beer, a weight range of 86–229 lbs, no mobility impairments, and not being pregnant or nursing.

###### a: EXCLUSION CRITERIA

Participants were excluded for prior substance use disorder treatment, medical conditions, medications, or alcohol flushing. Marijuana use was limited to three times per week in the past month, and no other drug (except marijuana) was allowed in the past week. Eligible participants abstained from alcohol and marijuana 24 hours before the session and from food (4 hours) and water (2 hours) prior. Transportation was provided for safety. In total, 121 participants met the criteria and provided usable data.

#### 3) STUDY PROCEDURES

##### a: BASELINE MEASUREMENTS

Participants signed a consent form and underwent ID verification, weight measurement, pregnancy tests (for women), and drug screening. Initial BAC was measured using an AlcoSensor IV Breathalyzer (Intoximeters Inc., St. Louis, MO). Ineligible participants received compensation but did not proceed. Eligible participants completed interviews, baseline walking tasks, and Field Sobriety Tests (FSTs), with BAC recorded before alcohol administration.

##### b: ALCOHOL ADMINISTRATION PROTOCOL

Participants consumed Hurricane Beer (8.1% alcohol) in doses adjusted to body weight—4 oz for males over 150 lbs and females over 160 lbs, 3 oz otherwise. BAC was checked 15 minutes after the first dose, with subsequent adjustments based on BAC trajectory. To avoid measurement interference, participants rinsed their mouths before breathalyzer tests. BAC monitoring continued until a peak of 0.10, after which they were allowed to eat. Walking tests and BAC checks were repeated at 7-minute intervals during the BAC reduction phase.

#### 4) WALKING EXPERIMENT PROTOCOL

##### a: EXPERIMENTAL SETUP

Walking tasks were conducted on a 75-foot marked hospital corridor path. Participants started at the midpoint, walked to one end, turned, walked to the other end, and returned to the starting point. Each trial lasted 45–60 seconds.

##### b: DATA COLLECTION DEVICES

Gait data were recorded using a Google Pixel XL smartphone and an LG Watch Sport running the Alcogait app. The smartphone, secured via a Velcro belt at the left-back pocket, and the smartwatch, worn on the left wrist, captured accelerometer and gyroscope readings. Participant details (ID, gender, age, height, weight) were entered before each session.

### B. EQUATIONS

Key metrics used in the evaluation are defined below:

(22)
$$\text{Accuracy}\;=\frac{TP\;+\;TN}{TP\;+\;TN\;+\;FP\;+\;FN}$$

(23)
$$F1=\frac{2\;\times \;\text{Precision}\;\times \;\text{Recall}\;}{\;\text{Precision}\;+\;\text{Recall}\;}$$

(24)
$$AUC=\int_{0}^{1}\;TPR(FPR)dFPR$$

(25)
$$\text{Specificity}\;=\frac{TN}{TN\;+\;FP}$$

(26)
$$\text{Recall}\;(TPR)=\frac{TP}{TP\;+\;FN}$$

(27)
$$\text{Precision}=\frac{TP}{TP\;+\;FP}$$

### C. SUPPLEMENTARY FIGURES AND TABLES

#### 1) ADDITIONAL ATTENTION VISUALIZATIONS

##### a: ATTENTION OVERLAY

Supplementary Figure S1 shows the direct overlay of attention patterns for both classes. The near-continuous green shading confirms that sober samples systematically require higher attention (mean: 0.074 vs. 0.064), supporting the finding that intoxicated patterns are more stereotyped and easier to classify. This distributed attention difference across the entire sequence suggests global pattern complexity differences rather than isolated critical events.

##### b: ATTENTION DIFFERENCE

Supplementary Figure S2 plots the attention gap as a direct subtraction (Intoxicated - Sober). The entirely negative curve (mean Δ=-0.010) provides mathematical confirmation that sober patterns require 13.5% higher attention. The oscillatory pattern (period ≈ 150–200 timesteps) reflects underlying gait cycle periodicity, with larger gaps at critical balance control phases. The consistent directional bias demonstrates robust, systematic complexity differences between classes.
